# SopB-Mediated Recruitment of SNX18 Facilitates *Salmonella* Typhimurium Internalization by the Host Cell

**DOI:** 10.3389/fcimb.2017.00257

**Published:** 2017-06-15

**Authors:** David Liebl, Xiaying Qi, Yang Zhe, Timothy C. Barnett, Rohan D. Teasdale

**Affiliations:** ^1^Institute for Molecular Bioscience, The University of QueenslandBrisbane, QLD, Australia; ^2^Australian Infectious Diseases Research Centre, The University of QueenslandBrisbane, QLD, Australia; ^3^School of Chemistry and Molecular Biosciences, The University of QueenslandBrisbane, QLD, Australia; ^4^School of Biomedical Sciences, The University of QueenslandBrisbane, QLD, Australia

**Keywords:** *Salmonella*, macropinocytosis, sorting nexin, phosphoinositide, host-pathogen interaction

## Abstract

To invade epithelial cells, *Salmonella enterica* serovar Typhimurium (*S*. Typhimurium) induces macropinocytosis through the action of virulence proteins delivered across the host cell membrane via a type III secretion system. We show that after docking at the plasma membrane *S*. Typhimurium triggers rapid recruitment of cytosolic SNX18, a SH3-PX-BAR domain sorting nexin protein, to the bacteria-induced membrane ruffles and to the nascent *Salmonella*-containing vacuole. SNX18 recruitment required the inositol-phosphatase activity of the *Salmonella* effector SopB and an intact phosphoinositide-binding site within the PX domain of SNX18, but occurred independently of Rho-GTPases Rac1 and Cdc42 activation. SNX18 promotes formation of the SCV from the plasma membrane by acting as a scaffold to recruit Dynamin-2 and N-WASP in a process dependent on the SH3 domain of SNX18. Quantification of bacteria uptake revealed that overexpression of SNX18 increased bacteria internalization, whereas a decrease was detected in cells overexpressing the phosphoinositide-binding mutant R303Q, the ΔSH3 mutant, and in cells where endogenous levels of SNX18 were knocked-down. This study identifies SNX18 as a novel target of SopB and suggests a mechanism where *S*. Typhimurium engages host factors via local manipulation of phosphoinositide composition at the site of invasion to orchestrate the internalization process.

## Introduction

To gain entry into non-phagocytic epithelial cells, various viral and bacterial human pathogens co-opt the endocytic machinery by modulation of phosphoinositide metabolism (Pizarro-Cerda and Cossart, 2004) or actin cytoskeleton (Haglund and Welch, 2011). One of the most efficient routes for pathogens to enter the host cells is macropinocytosis, a distinct form of endocytosis characteristic for its non-selective and high turnover uptake of extracellular fluid into macropinosomes (Lim et al., 2008; Kerr et al., 2010).

*Salmonella* are facultative intracellular Gram-negative bacteria, which infect and replicate within both epithelial cells and macrophages. To invade epithelial cells, *Salmonella* induces macropinocytosis at the site of entry (Francis et al., 1993) by translocating a set of effector proteins into the host cell cytoplasm via a type III secretion system (T3SS) encoded by *Salmonella* pathogenicity island 1 (SPI1). Interactions between the translocated effector proteins and host cell targets result in orchestrated manipulation of phosphoinositide signaling, Rho-GTPase function and actin cytoskeleton remodeling that promotes internalization of the bacteria into a membrane-bound organelle, termed the *Salmonella*-containing vacuole (SCV) (Haraga et al., 2008). Although it has been reported that the biogenesis and release of the nascent SCV from the plasma membrane is mediated by the virulence protein SopB (Terebiznik et al., 2002), most likely in cooperation with SopD (Bakowski et al., 2007), the host-cell factors involved in this process remain largely unknown.

*Salmonella enterica* serovar Typhimurium (*S*. Typhimurium) has been used as a model organism to study the role of phosphoinositides in bacteria-mediated manipulation of the host endocytic trafficking pathways (Kerr et al., 2010). The phosphoinositides are key regulators of the actin cytoskeleton and membrane trafficking and are indispensable for macropinocytosis and maturation of the SCV (Kerr et al., 2012). Specific membrane bound phosphoinositides are essential for recruitment of sorting nexins, a family of Phox homology (PX) domain containing proteins that orchestrate membrane trafficking, cargo sorting, and endosomal recycling in coordination with other effectors including RabGTPases (Teasdale and Collins, 2012). Several sorting nexins (SNX), including SNX1 and SNX3, have been functionally linked to membrane tubulation, remodeling and maturation of the SCV during early stages of *S*. Typhimurium infection (Bujny et al., 2008; Braun et al., 2010) and our earlier studies also shown that SNX1 and SNX5 are essential for membrane recycling and macropinosome biogenesis in non-infected cells (Bryant et al., 2007; Lim et al., 2008). Our recent screen of SNX-PX-BAR proteins involved in macropinocytosis identified another member of sorting nexin family, SNX18, which significantly increased the fluid phase uptake when overexpressed in epithelial cells (Wang et al., 2010).

The function of SNX18 is still not well-understood. Recent studies proposed that SNX18 might function as a paralog of SNX9 that is engaged in endosome formation and scission from the plasma membrane by coupling phosphoinositide recognition to actin assembly (Soulet et al., 2005; Pylypenko et al., 2007; Park et al., 2010). SNX18 and SNX9 are homologs with an identical SH3-PX-BAR domain modular structure and some conserved binding partners (Haberg et al., 2008; Park et al., 2010). However, variations between the SNX9 and SNX18 expression profile within different cell lines (Park et al., 2010), tissues (Haberg et al., 2008), or embryonic stages (Nakazawa et al., 2011), and differences in their affinity to particular membrane bound phosphoinositides (Haberg et al., 2008; Yarar et al., 2008) suggest that SNX18 and SNX9 may have evolved to perform different functions.

In this study, we investigate the function of SNX18 in *Salmonella*-driven exploitation of macropinocytosis during invasion of epithelial cells. We demonstrate that *S*. Typhimurium recruits SNX18, N-WASP and Dynamin-2 to the site of invasion through the phosphatidylinositol phosphatase activity of the T3SS effector SopB. We propose that modulation of phosphoinositides at the site of *S*. Typhimurium invasion recruits SNX18 as a scaffold to organize the molecular machinery required for formation and scission of the nascent SCV from the cell surface, which increases the efficiency of bacteria internalization.

## Materials and methods

### Antibodies and reagents

The following primary antibodies were used: Rabbit polyclonal anti-SNX18 IgG (AbCam, ab99035); mouse monoclonal anti-myc (Cell Signaling Technology, 2272); Rabbit polyclonal anti-β-Tubulin (LI-COR Biosciences, 926-42211); mouse monoclonal anti-α-tubulin Clone DM 1A (Sigma Aldrich, T9026), anti-α1 Na+/K+ ATPase antibody (ab7671, Abcam), mouse monoclonal anti-LPS (Abcam, ab 8274); Mouse anti-Phospho-Tyrosine antibody, clone 4G10 (Millipore, 05-1050); mouse monoclonal anti-HA (Covance); Rabbit monoclonal anti-Akt (pan) C67E7 (Cell Signaling, 4691); and anti-phospho-Akt (Ser473) D9E (Cell Signaling, 4060). Goat anti-mouse coupled to Alexa Fluor 405, 546 or 647 (Invitrogen) were used as secondary antibodies for immunofluorescence. The IRDye800CW goat anti-mouse IgG; IRDye800CW donkey anti-rabbit IgG; IRDye680LT goat anti-rabbit IgG; and IRDye680LT donkey anti-mouse IgG were purchased from LI-COR Biosciences. Phalloidin-Alexa Fluor 635, fixable analog of lipophilic membrane stain FM 4-64FX and Wheat germ agglutinin (WGA) coupled to Alexa Fluor 647 were from Invitrogen (A34054, F34653, and W324666). Selective inhibitor of Rac1-GEF interaction NSC 23766 was purchased from Tocris Bioscience and the Akt1/2 kinase inhibitor from Sigma Aldrich.

### Plasmids and constructs

The Dynamin-2 encoding construct pCBDYN2-HA was obtained from Dr. S.L. Schmid (Scripps Research Institute, La Jolla, CA). The 2xFYVE-EGFP construct was generated as described by Pattni et al. (2001) and provided by F. Meunier (University of Queensland, Australia). The construct expressing EGFP-tagged PH domain of Akt was a gift from Prof. C. Mitchell (Monash University, Australia) and the PH domain of TAPP1 (aa 95–400) for generation of EGFP-TAPP1-PH was a gift from Dr. S. Dowler (MRC, University of Dundee, Dundee, UK). The following expression vectors have been described previously: The pEGFP-C1-N-WASP (Lommel et al., 2001), the pcDNA3 expression vectors encoding constitutively active Rac1 or Cdc42 and dominant negative Rac1 or Cdc42 (Wang et al., 2005), the pEGFP-FERM (Marion et al., 2011), the pEGFP-SNX18 and pEGFP-SNX18:ΔSH3 (Wang et al., 2010), the Btk-PH-GFP (Varnai et al., 1999). The LifeAct-Ruby construct was a gift Roland Wedlich-Söldner, (Max Planck Institute of Biochemistry, Germany).

The pEGFP-SNX18:R303Q was generated by site-directed mutagenesis from the full length pEGFP-SNX18. Cloning of N-terminal-myc-tagged *S*. Typhimurium effectors was performed by ligation-independent cloning (LIC) (Aslanidis and de Jong, 1990) into the plasmid pcDNA3.1-nMyc-LIC, constructed as follows: A DNA cassette encoding a Myc tag, LIC sequences, chloramphenicol acetyl transferase (*cat*), and *ccdB* was constructed by PCR using primers N-Myc-catccdB-NheI-S and catccdB-ApaI-A and Reading Frame Cassette A template DNA from the Gateway Vector Conversion System (Life Technologies); The resulting PCR product was digested with NheI-ApaI and ligated into NheI-ApaI-digested pcDNA3.1(+). The resulting plasmid, pcDNA3.1-nMyc-LIC, was maintained in *E. coli ccdB* Survival™2 T1^R^ cells (Life Technologies). For LIC reactions, pcDNA3.1-nMyc-LIC was digested with EcoRV and treated with T4 DNA polymerase in the presence of dCTP to generate linearized vector with single-stranded DNA overhangs. The genes encoding individual *S*. Typhimurium effectors were amplified by PCR using primers which incorporated 5' and 3' LIC sequences and *S*. Typhimurium SL1344 template DNA. Each PCR product was treated with T4 DNA polymerase in the presence of dGTP to generate single-stranded overhangs. The polymerase-treated vector and effector DNA fragments were combined on ice and transformed into chemically-competent *E. coli* DH5α. Vectors encoding Myc-tagged phosphatase inactive SopB mutants SopB:C460S, R466A, and K528A were constructed by PCR amplification using pcDNA3.1 (+) vector encoding Myc-tagged SopB (wild type) as a template. All mutants were constructed using the QuikChange XL-site directed mutagenesis kit (Stratagene) according to manufacturer's instructions, and sequences were confirmed by direct DNA sequencing at AGRF (Australian Genome Research Facility). All primers used in this study are listed in Table 1.

**Table 1:** Primers used in this study.

**Primer**	Sequence^*^
N-Myc-catccdB-NheI fwd	5'-GCATGCTAGCCACC**ATGGAGCAGAAGC****TGATAAGTGAGGAGGAT****ATC**AGGCA CCCCAGGCTTTACACTTTATGCTTC-3'
catccdB-ApaI rev	5'-GCATGGGCCCGACATGAAGGTTAGGGATATCGACCTGCAGACTGGCTGTGTATA AG-3'
sipA fwd	5'-TGATAAGTGAGGAGGATCTGGTTACAAGTGTAAGGACTCAGC-3'
sipA rev	5'-ATGATGGTTAGGGATCTTAACGCTGCATGTGCAAGCCATC-3'
sipC fwd	5'-TGATAAGTGAGGAGGATCTGTTAATTAGTAATGTGGGAATAAATC-3'
sipC rev	5'-ATGATGGTTAGGGATCTTAAGCGCGAATATTGCCTGCGAT-3'
sopB fwd	5'-TGATAAGTGAGGAGGATCTGCAAATACAGAGCTTCTATCACTC-3'
sopB rev	5'-ATGATGGTTAGGGATCTCAAGATGTGATTAATGAAGAAATGCCTTTTACTG-3'
sopD fwd	5'-TGATAAGTGAGGAGGATCTGCCAGTCACTTTAAGCTTCGGTAATCATC-3'
sopD rev	5'-ATGATGGTTAGGGATCTTATGTCAGTAATATATTACGACTGCACCCATC-3'
sopE2 fwd	5'-TGATAAGTGAGGAGGATCTGACTAACATAACACTATCCACCCAGCAC-3'
sopE2 rev	5'-ATGATGGTTAGGGATCTCAGGAGGCATTCTGAAGATACTTATTC-3'
sptP fwd	5'-TGATAAGTGAGGAGGATCTGCTAAAGTATGAGGAGAGAAAATTGAATAATTTA ACGTTG-3'
sptP rev	5'-ATGATGGTTAGGGATCTCAGCTTGCCGTCGTCATAAG-3'
SopB:C460S fwd	5'-GGTACCCGCCTGGAATAGTAAAAGCGGCAAAG-3'
SopB:C460S rev	5'-CTTTGCCGCTTTTACTATTCCAGGCGGGTACC-3'
SopB:R466A fwd	5'-CCTGGAATTGTAAAAGCGGCAAAGATGCTACAGGGATGATGG-3'
SopB:R466A rev	5'-CCATCATCCCTGTAGCATCTTTGCCGCTTTTACAATTCCAGG-3'
SopB:K528A fwd	5'-GGGCGGGAAACAAAGTAATGGCAAATTTATCGCCAGAGGTGC-3'
SopB:K528A rev	5'-GCACCTCTGGCGATAAATTTGCCATTACTTTGTTTCCCGCCC-3'
SNX18:R303Q fwd	5'-CCAGGTGCCCGTGCACAGGCAATATAAGCACTTCGATTGG-3'
SNX18:R303Q rev	5'-CCAATCGAAGTGCTTATATTGCCTGTGCACGGGCACCTGGG-3'
sopB/sopB:C460S fwd	5'-GCATGAATTCTATTCAGGAATATTAAAAACGCTATGCAAATACAGAGCTTCTATCACTC-3'
sopB/sopB:C460S rev	5'-GCATCTCGAGTACCTCAAGACTCAAGATGTGATTAATGAAGAAATGCCTTTTACTG-3'

*fwd, Forward; rev, reverse*.

* *Sequences added to the 5' end of primers to allow LIC are underlined. The sequence encoding the Myc tag is in bold font*.

For complementation of SopB in *S*. Typhimurium Δ*sopB* mutant bacteria, the coding sequence of the wild type *sopB* and that of the C460S mutant of *sopB* were amplified by PCR using pcDNA3.1 (+) vector encoding Myc-tagged SopB (wild type) or Myc-tagged C460S mutant of SopB as templates. Corresponding primers used for the PCR are listed in Table 1. The PCR products were digested with EcoRI and XhoI and subcloned into pWSK29 vector (GenBank: AF016889.1).

### Cell culture, transfections, and generation of SNX18 knockdown

Human epithelial HEK293 cells (CRL-1573) and mouse macrophages RAW264.7 (TIB-71) were grown in complete DMEM medium (Life technologies) supplemented with 10% (v/v) FCS. Cells were transfected using Lipofectamine 2000 (Invitrogen). For stable expression, transfected cells were selected with 400 μg/ml Geneticin (G418), and cell lines were generated by limit dilution. To generate the shRNA-mediated knockdown of SNX18, the pGIPZ-shRNAmir clones (V2LHS_184681, V2LHS_37858, V2LMM_58706) complementary to human SNX18 were obtained from Thermo Scientific. HEK293 cells were transfected with pGIPZ constructs using Lipofectamine 2000 (Invitrogen) and non-silencing shRNA was transfected as a control. Cells were split 24 h post transfection and selected in 1 μg/ml puromycin for 3 or more days before SNX18 protein levels were tested by western blot. Cells were then transfected as above, selected with 1 μg/mL puromycin for 7 days to generate stable cell lines. Cells stably expressing non-silencing shRNA were used as a control knockdown.

### Bacteria strains and infections

Wild type *S*. Typhimurium strain SL1344 expressing pBR-mRFP.1 (Birmingham et al., 2006) or pFPV25.1-GFP (Knodler et al., 2005) were used in this study. The isogenic Δ*sopB* mutant has been described earlier (Steele-Mortimer et al., 2000) and provided by Dr. N. Brown (Department of Microbiology and Immunology; University of Melbourne; Australia). The *S*. Typhimurium SL1344 mutants Δ*invA* (SPI1-T3SS deficient) and Δ*ssaR* (SPI2-T3SS deficient) were provided by Prof. R. Strugnell (University of Melbourne, Australia) (Kupz et al., 2012). Where non-fluorescent bacteria were utilized, the mouse monoclonal anti-LPS antibody (Abcam) was used for immunofluorescent detection. To prepare invasive (SPI1-T3SS activated) bacteria, the overnight culture was subcultured 1:60 in LB medium and grown for another 4 h to reach late log phase. Bacteria were washed three-times in Hanks buffered salt solution (HBSS) and diluted in serum-free DMEM medium (for immunofluorescence) or in CO_2_-independent imaging medium (Invitrogen) for live imaging. For complementation of SopB in *S*. Typhimurium Δ*sopB* mutant bacteria, the sequence verified plasmids were transformed into electrocompetent *S*. Typhimurium Δ*sopB* mutant bacteria by electroporation using Bio Rad Gene Pulser II Electroporation System and positive clones of complemented *S*. Typhimurium were selected using kanamycin.

### Electron microscopy

The HEK293 cells expressing EGFP-SNX18 were infected (or mock infected) with *S*. Typhimurim (SL1344) for 10 min, fixed with 4% (w/v) formaldehyde, and 0.1% glutaraldehyde (w/v) in 0.1 M phosphate buffer and embedded in 10% (w/v) gelatin (Sigma). Solidified gelatin blocks were infiltrated with 2.3 M sucrose, mounted on aluminum pins and frozen in liquid nitrogen. Ultrathin sections were cut on Leica EM UC6 cryotome, collected on a drop of sucrose–methylcellulose and transferred on carbon-coated CuPd EM grids with formvar film. EGFP-SNX18 was detected on sections by rabbit anti-GFP antibody (Molecular Probes) followed by protein A coupled with 10 nm gold (CMC Utrecht). Grids were contrasted, embedded in mixture of uranyl acetate–methylcellulose and analyzed with JEOL 1011 electron microscope operating at 80 kV. Micrographs were acquired by microscope-mounted CCD camera and ImageJ software (version 1.46a) was used for contrast adjustment and cropping of the final images. Manual quantification of gold marker localization was performed on micrographs by scoring each gold particle in a set of images for localization in plasma membrane or in cytosol.

### Subcellular fractionation and western blotting

HEK293 cells expressing EGFP-tagged SNX18 were serum starved overnight before bacterial infection at MOI = 100 or mock infection. At 10 min post infection, cells were washed twice with ice-cold PBS and harvested for subcellular fractionation using a modification of the protocol described by Piper et al. (1991). Briefly, cells were resuspended in HES buffer (20 mM HEPES, pH 7.4, 0.25 mM sucrose, 1 mM EDTA, 10 mM sodium pyrophosphate, 30 mM sodium vanadate, 0.5 mM AEBSF and protease inhibitor cocktail) and subjected to homogenization by using 22G × 3/4 needle. Resulting suspensions were centrifuged at 17,200 g for 20 min to generate crude plasma membrane (PM) fraction and a fraction containing cytosol and microsomes. The cytosol fraction was then purified by microsome sedimentation at 180,000 g for 60 min. Crude PM fractions were washed once and resuspended in HES buffer, and then layered onto 1.12 M sucrose cushion, containing 20 mM HEPES, pH 7.4, 1.12 M sucrose and 1 mM EDTA, followed by centrifugation at 100,000 g for 1 h to yield a white interface, which contains the purified PM fraction. The white interface was mixed with HES buffer, and centrifuged at 125,000 g for 30 min to pellet the purified PM fraction. For protein electrophoresis, 30 μg of total protein of whole cell lysate or cytosol fractions or 15 μg of total protein of the plasma membrane fractions were loaded on to 8% (w/v) SDS-PAGE gel and transferred onto a PVDF membrane (Immobilon-FL, Millipore) according to manufacturer's instructions. Western immunoblots were performed with anti-SNX18, anti-α-Tubulin and anti-Na+/K+ ATPase primary antibodies in Odyssey blocker, followed by IRdye800 anti-rabbit secondary antibody, and membranes were scanned using Odyssey Infrared Imaging System (LI-COR Biosciences) according to manufacturer's instructions. The integrated intensity of each band of interest was measured by Odyssey software.

### AKT inhibitor treatment and *S*. Typhimurium infection

Overnight serum-starved HEK293 cells were pre-treated with 10 μM AKTi1/2 (Sigma-Aldrich) for 30 min before infection with *S*. Typhimurium (wild type or Δ*sopB*). At 10 min post infection, cells were washed with ice-cold PBS and harvested in lysis buffer (50 mM HEPES (pH 7.4), 1% (v/v) Triton x-100, 150 mM sodium chloride, 1 mM EDTA, 10 mM sodium pyrophosphate, 30 mM sodium vanadate, 0.5 mM AEBSF and protease inhibitor cocktails). Cell lysates were cleared by centrifugation at 16,000 g for 10 min and protein concentrations were quantified by BCA protein assay. Equal amounts of cell lysates were subjected to SDS-PAGE and immunoblotting, staining with anti-phospho Ser^473^ Akt, anti-Akt, or anti-Tubulin antibodies, followed by IRdye800 anti-rabbit or IRdye680 anti-mouse secondary antibodies. Fluorescence intensities were detected using the LI-COR Biosciences Odyssey Infrared Imaging system.

### Immunofluorescence

For immunofluorescence, cells were grown on poly-L-lysine (PLL)-coated glass coverslips, fixed with 4% (w/v) formaldehyde in 250 mM HEPES and permeabilized with Triton X-100 (0.25% (v/v) in PBS) before incubation with antibodies. Where non-fluorescent bacteria were used, cell permeabilization was required prior to bacteria immunodetection with anti-LPS antibody. For detection of extracellular and/or incompletely internalized mRFP-expressing bacteria, cells were incubated with the anti-LPS antibody prior to cell permeabilization. Cells on coverslips were incubated with primary antibodies in 0.25% (w/v) BSA in PBS for 1 h, followed by wash in PBS and 1 h incubation with secondary antibodies with or without DAPI and fluorescent Phalloidin (all in 0.25% (w/v) BSA in PBS). After washing in PBS, coverslips were rinsed in water and mounted on glass slides using Dako Fluorescent Mounting Medium. Fluorescent images were acquired using confocal microscope Zeiss LSM 710 with Plan-Apochromatic 63x/1.4 Oil DIC objective, operated by ZEN2000 acquisition software, or using fluorescence microscope Olympus DP-71 with Plan-Apochromatic 10x/0.40 and 20x/0.75 objective, equipped with 12 Mp Color Camera and images acquired using DP Capture and DP Manager software.

### Live cell imaging

Cells were grown on glass bottom chamber slides (Lab-Tek, Thermo Scientific), and the culture medium was replaced with CO_2_-independent medium (Invitrogen) prior to imaging. To examine the interaction between bacteria and the host cells from the onset of infection, invasive (SPI1-T3SS-induced) bacteria were pre-diluted in CO_2_-independent medium and added directly to the chamber slide “on stage” during time-lapse imaging. The confocal z-stacks or time-lapse of single confocal sections were acquired typically during the first 10 min of infection at a frequency of 6 s between frames and at the maximal resolution of 70 nm of pixel size. A Zeiss LSM 710 FCS inverted confocal microscope operated by ZEN2009 acquisition software and equipped with a temperature controlled incubation chamber and Plan-Apochromat 63x/1.4 Oil DIC objective was used for imaging.

### Image processing and image analysis

ImageJ software (version 1.46a) was used for fluorescent channels splitting or merging, image area cropping, orthogonal or maximum z-stack projections, time stamper insertions, montage of selected frames, RGB line profiling, and brightness and contrast adjustment of raw acquisition images where required. To analyze the kinetics of protein recruitment or depletion, the increase or decrease of EGFP and mCherry fluorescence intensity was assessed by stack-measurement of integrated fluorescence density of selected ROI (all identical size) after the RGB image was split in separate eight-bit fluorescent channels. Absolute values were normalized to frame 1 for both channels to demonstrate the fold of fluorescence increase/decrease over time of the sequence. Corresponding ROIs in non-infected cell (on the same movie) were selected as a control for fluorescence fluctuations and bleaching. The co-localization between SNX18 and phosphoinositide binding probes has been analyzed by co-localization highlighter plugin in (ImageJ) and the total co-localization area in each time point of the movie was quantified by particle analysis. Co-localization between SNX18 and Dynamin-2 or N-WASP within bacteria-containing membrane ruffles was quantified by co-localization threshold plugin in ImageJ. From 10 to 20 cells or regions of interest (ROI) per each sample were analyzed. To demonstrate an increase or decrease of co-localization relative to controls, statistics were calculated from values that represent the percentage of voxels which have both channel 1 and channel 2 intensities above threshold, expressed as a percentage of the total number of pixels in the image.

### Image-based quantification of macropinocytosis and bacterial uptake

The rate of macropinocytosis was assessed by fluorescent-dextran uptake assay. Briefly, cells were grown on PLL-coated coverslips and starved O/N in FCS-free DMEM medium before adding 100 ug/ml of anionic, fixable Dextran (MW 10000) conjugated to Alexa Fluor 647 with 10 ng/ml of EGF. After 10 min of dextran uptake, cells were fixed with 4% (w/v) formaldehyde in 250 mM HEPES and mounted on coverslips for imaging using fluorescent mounting medium (Dako). For the bacterial uptake assay, stable SNX18 knockdown cells (RFP-positive) and SNX18 overexpressing cells (EGFP-positive) were grown as above and infected with EGFP or RFP-expressing *S*. Typhimurium respectively, at an MOI = 10. At 10 min post infection, cells were washed, fixed and mounted for imaging as described above. Macropinosomes and intracellular bacteria were quantified using ImageJ software for image analysis as described previously (Wang et al., 2010). Briefly, RGB images were split to eight-bit channels (EGFP and RFP), subjected to segmentation by thresholding, cell mask subtraction by image calculator (to exclude extracellular dextran aggregates or non-internalized bacteria) and particle analysis function was then used to measure counts, integrated density and area of spots. Original images were acquired under the same conditions for all samples, batch processing was performed using ImageJ Macro and about 250~300 cells per sample were quantified for statistical evaluation.

## Results

### SNX18 is recruited to the plasma membrane during *S*. Typhimurium invasion

**Figure 1:**
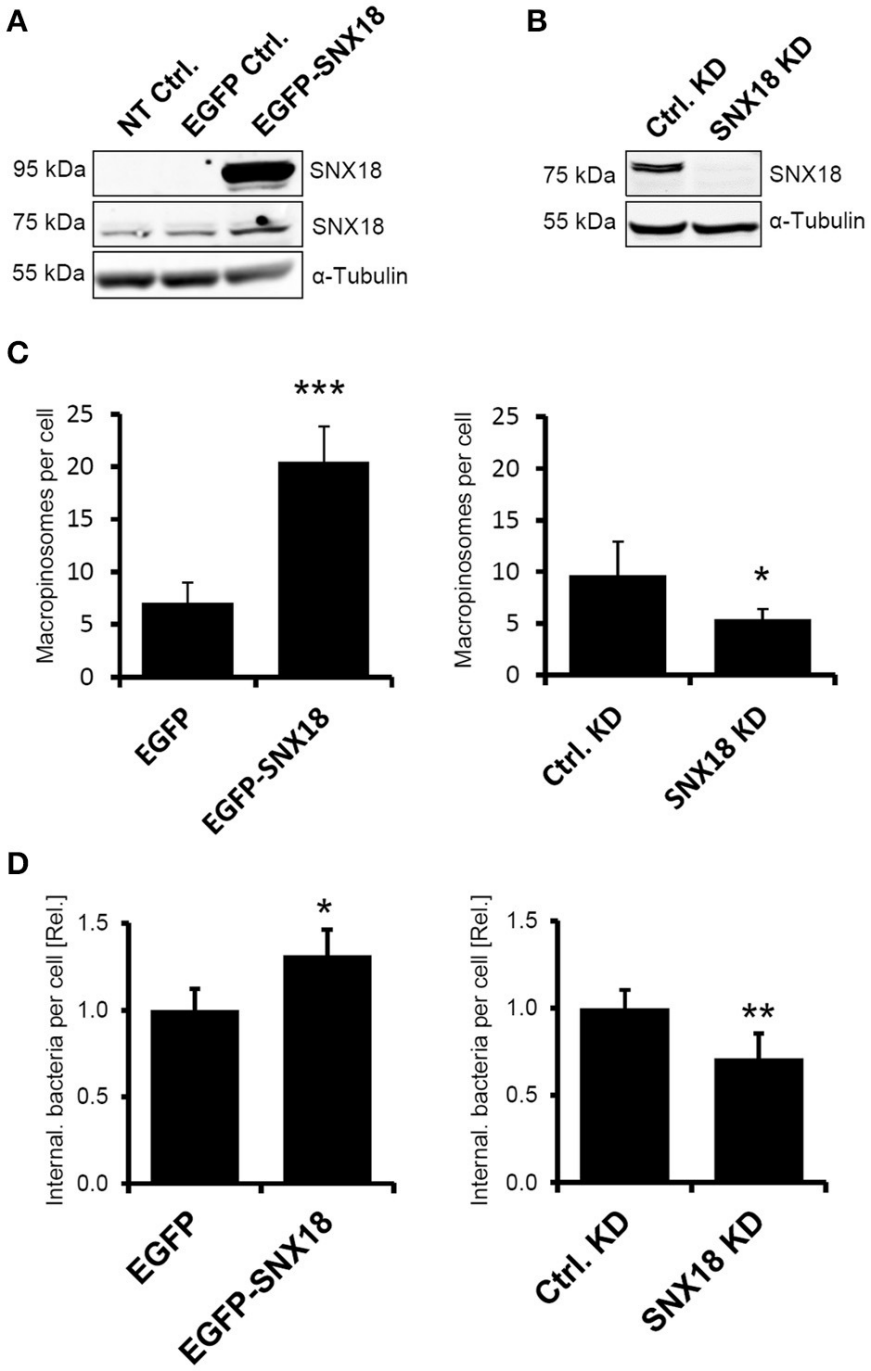
SNX18 functions in macropinocytosis and *S*. Typhimurium internalization. **(A)** Protein levels of endogenous SNX18 (75 kDa) and EGFP-SNX18 (95 kDa) in non-transfected HEK293 cells and in cells overexpressing EGFP or EGFP-SNX18 were detected by western blot using anti-SNX18 antibody. **(B)** Protein levels of endogenous SNX18 in cells 6 days post induction of shRNA-mediated knockdown and in control (non-induced) cells. α-Tubulin was used as a loading control. **(C)** The rate of macropinocytosis in cells overexpressing SNX18 (left) and in SNX18 knockdown cells (right) assessed by fluorescent dextran uptake assay in EGF-treated HEK293 cells. Numbers of dextran-positive macropinosomes per cell were counted by image analysis. The average values in control cells were normalized to one to demonstrate the fold change in EGFP-SNX18 expressing cells and SNX18 knockdown cells. Between 250 and 300 cells per sample were analyzed. Bars indicate the mean + standard deviation between three experiments. ^***^p < 0.001, ^*^p < 0.05, *t*-test. **(D)** Efficiency of bacteria internalization in cells overexpressing SNX18 (left) and in SNX18 knockdown cells (right). The amounts of intracellular bacteria per cell were quantified at 10 min post infection using image analysis with exclusion of extracellular bacteria (labeled by anti-LPS antibody). The average values in control cells were normalized to one to demonstrate the fold change in EGFP-SNX18 expressing cells and SNX18 knockdown cells. Bars indicate the mean + standard deviation between three experiments, ^*^p < 0.05, ^**^p < 0.01, *t*-test.

We reported recently (Wang et al., 2010) that SNX18 was the most potent of the three SH3-PX-BAR sorting nexins in increasing the EGF-stimulated macropinocytosis when transiently overexpressed in epithelial cells. Because macropinocytosis is targeted by *S*. Typhimurium, we first aimed to determine the role of SNX18 in *S*. Typhimurium invasion of epithelial cells by assessing the rate of macropinocytosis and efficiency of bacterial internalization in human embryonic kidney epithelial cells (HEK293) overexpressing EGFP-SNX18 and in HEK293 cells where endogenous levels of SNX18 were reduced by inducible shRNA-mediated knockdown. In EGFP-SNX18 transfected cells the SNX18 expression levels (Figure 1A) were ~20-fold higher than endogenous levels (data not shown), while the endogenous levels of SNX18 in the stable SNX18 shRNA cell line were below detection 6 days post induction of shRNA expression (Figure 1B).

To analyze the effect of SNX18 expression levels on macropinocytosis, we measured the rate of macropinocytosis in each cell line by dextran uptake assay using fluorescently labeled dextran. Quantification of dextran-positive macropinosomes (integrated fluorescent density) in EGF-treated cells after 10 min of dextran uptake revealed 3-fold increase in cells expressing EGFP-SNX18 in comparison to controls, and a 2-fold decrease in SNX18 knockdown cells relative to control knockdown (Figure 1C).

Next we investigated whether overexpression or knockdown of SNX18 affects internalization of *S*. Typhimurium. Cells were infected with mRFP-expressing *S*. Typhimurium (SL-mRFP) and the amount of fully internalized bacteria was determined at 10 min post infection by quantitative fluorescence microscopy. Extracellular bacteria were differentiated from internalized bacteria by immunostaining of non-permeabilized cells with anti-LPS antibody. The amount of internalized bacteria was 32 ± 6% higher in EGFP-SNX18 expressing cells when compared to EGFP-expressing control cells, while knockdown of SNX18 decreased the amount of intracellular bacteria by 29 ± 9% relative to controls (Figure 1D). Thus, although depletion of SNX18 did not completely abolish macropinocytosis or bacteria internalization, modulation of SNX18 levels resulted in significant impact on both macropinocytosis and *S*. Typhimurium internalization. In order to minimize the influence of passive uptake of bacteria from the media, we analyzed short-lasting but synchronized (simultaneous) invasion at relatively high MOI, rather than prolonged invasion at low MOI, that would imply consecutive (and potentially passive) invasion events. For this we reduced bacteria adhesion/invasion time to 10 min only and optimized the MOI so that at end point of our invasion assay each cell internalized on average 1–3 bacteria (MOI = 10) for quantification assay.

**Figure 2:**
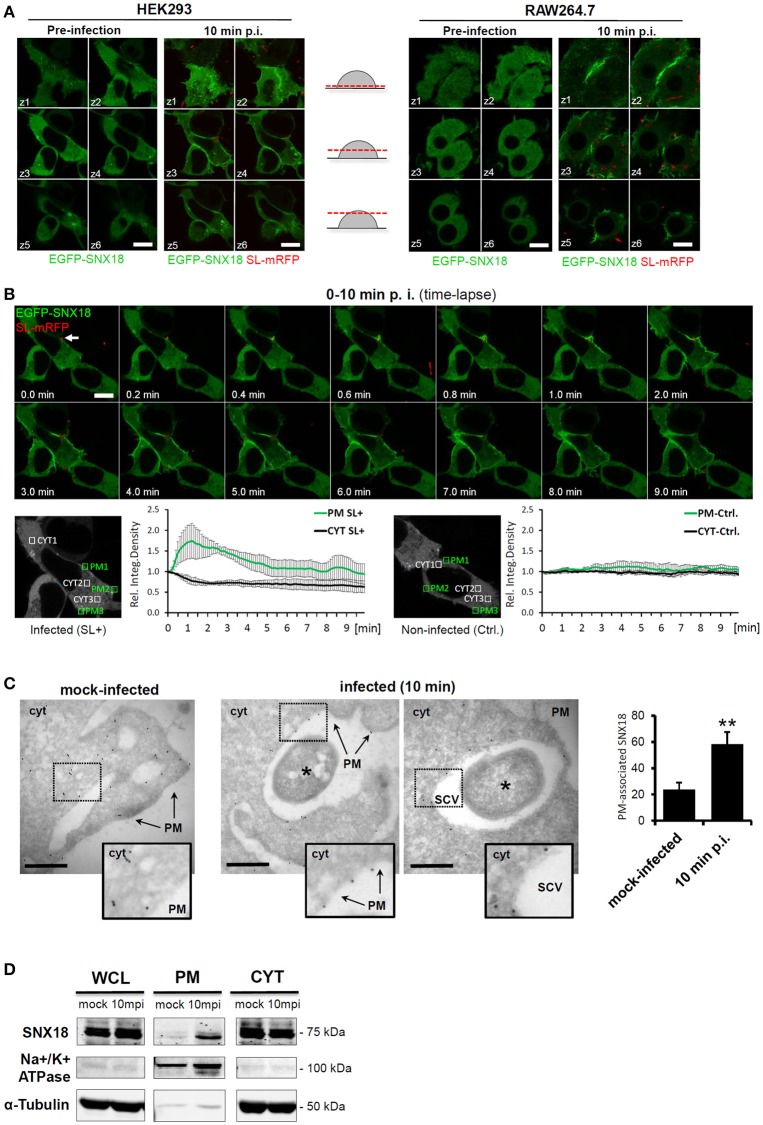
Cytosolic SNX18 is recruited to the site of bacterial invasion at the plasma membrane. **(A)** Within 10 min of infection, the cytosolic fraction of EGFP-SNX18 is recruited to the plasma membrane in both, epithelial cells (HEK293) and macrophages (RAW264.7). Shown is a montage of confocal z-stack sections through live cells expressing EGFP-SNX18 pre-infection and 10 min post infection with SL-mRFP. Bars = 10 μm. **(B)** Kinetic measurements of SNX18 relocation to the plasma membrane. Upper panel: Series of confocal images from a time-lapse acquisition of HEK293 cells expressing EGFP-SNX18 during 10 min of infection with SL-mRFP (initial docking site of bacteria indicated by an arrow); Lower panel: Quantification of fluorescence (relative integrated density) within selected ROI at plasma membrane and in cytosol of infected (left) and adjacent non-infected control cell (right). Absolute values of integrated density at T = 0 min post infection were normalized to 1 to demonstrate fold-increase/decrease in following time points. **(C)** Electron micrographs of frozen-hydrated sections through HEK293 cells with stable expression of EGFP-SNX18 fixed 10 min post infection (or mock-infection). The EGFP-SNX18 was detected by anti-GFP antibody followed by 10 nm Protein A-Gold. The marker of SNX18 localizes primarily to the cytosol (cyt) in mock infected cells, while upon infection the SNX18 associates mainly with the plasma membrane (PM) and the membrane of the nascent SCV. Bacteria indicated by asterisk. Bar = 1 μm. Quantification of gold markers associated with plasma membrane in mock-infected cells and cells 10 min p.i. as a percentage of total gold markers per each image analyzed. *N* = 10, *p* = 0.002 (*t*-test). **(D)** Endogenous SNX18 is also recruited to the plasma membrane during bacteria internalization: Whole cell lysate (WCL), plasma membrane (PM), and cytosolic (CYT) fractions from cells harvested at 10 min post infection (10 mpi) or mock-treatment (as a control). Endogenous SNX18 was detected on Western blot by anti-SNX18 antibody, PM fraction control Na+/K+ ATPase (absent in cytosol) by anti-α1 Na+/K+ ATPase antibody and cytosol fraction control Tubulin by anti-α-Tubulin antibody. ^*^p ≤ 0.05; ^**^p ≤ 0.01.

We next examined the subcellular localization and kinetics of EGFP-SNX18 in live HEK293 epithelial cells and RAW264.7 macrophages. Both of these cell types were selected as they undergo constitutive macropinocytosis with the majority of cells having numerous filopodia and also peripheral membrane regions exhibiting “ruffling.” This enables monitoring the capacity of *S*. Typhimurium to elevate the local recruitment of proteins like SNX18, within the modified plasma membrane regions. During transient expression in non-infected cells, EGFP-SNX18 localization varied with protein expression levels, where high levels led to formation of elongated, thickened and often branched membrane tubules described previously in cells transfected with myc-tagged PX-BAR construct of SNX18 (Haberg et al., 2008). Therefore, for further analysis we isolated cells expressing moderate levels of exogenous SNX18, where EGFP-SNX18 localized to the cytosol but a fraction was readily associated with endosomes and with short, endosome-derived tubules usually concentrated at the base of the cell where EGFP-SNX18 co-localized with fluorescent lipophilic vital stain FM4-64 (Figure S1). Upon infection with *S*. Typhimurium, we observed a dramatic relocation of EGFP-SNX18 from the cytosol to the plasma membrane within 10 min post infection. A similar extent of SNX18 relocation was detected in RAW264.7 macrophages (Figure 2A) and in other human epithelial lines, including HeLa, MCF-7, and A431 (not shown), suggesting that *Salmonella*-mediated engagement of SNX18 is utilized in multiple cell types including those that do not undergo constitutive macropinocytosis like HeLa cells.

To investigate the dynamics of SNX18 recruitment to the plasma membrane during *S*. Typhimurium internalization, we performed live imaging of EGFP-SNX18-expressing HEK293 cells within the first 10 min of infection. Following docking of bacteria to the plasma membrane at the site of invasion, we observed a rapid burst of SNX18 recruitment within membrane ruffles when compared with non-infected cells, where a transient increase of SNX18 was detected only on the nascent macropinosomes. (Figure 2B and Movie 1). To examine the kinetics of SNX18 recruitment during early stages of infection, we next measured the amount of EGFP-SNX18 at the plasma membrane and cytosol at various time points post infection by quantitating the fluorescence density profiles of selected regions of interest. As shown in Figure 2B, the amount of EGFP-SNX18 at the plasma membrane rapidly increased 1.7-fold within the first minute of infection while the concentration (fluorescence density) of the cytosolic pool of SNX18 within the same cell decreased. In contrast, insignificant changes in fluorescent density of EGFP-SNX18 in both, plasma membrane and cytosol were observed in non-infected cells.

To further examine the bacteria-induced recruitment of SNX18 to the plasma membrane at site of invasion, we infected (or mock-infected) the EGFP-SNX18 expressing cells and analyzed localization of this construct by electron microscopy using immunogold labeling on cryosections. In mock-infected cells, the gold-coupled marker of EGFP-SNX18 localized predominantly to the cytosol with only minor fraction (24 ± 4.5%) found in plasma membrane, while in infected cells, the majority (58 ± 5.5%) of the marker associated with the plasma membrane of bacteria-containing membrane ruffles and with the SCV during its closure at the cell periphery. In contrast, fully internalized SCV were devoid of SNX18 (not shown), suggesting that association of SNX18 with the SCV is only transient and occurs during the very early stage of the SCV formation (Figure 2C).

To confirm that also endogenous SNX18 is recruited to the plasma membrane the levels of cytosolic and membrane-bound SNX18 during bacteria internalization was then examined by cell fractionation and immunoblotting with anti-SNX18 antibodies. In agreement with the above results, the protein levels of membrane-bound endogenous SNX18 increased within the first 10 min of infection (Figure 2D) indicating that the presence of the EGFP-tag on SNX18 is not responsible for the induced recruitment to membranes observed during *S*. Typhimurium invasion in live cells.

We thus identified SNX18 within two fractions: a mobile, cytosolic fraction, and a membrane-bound fraction. Furthermore, we show that this ratio rapidly changes when SNX18 is recruited to the site of bacteria internalization. The kinetics of bacteria-induced SNX18 recruitment and the details of SNX18 localization within bacteria-containing membrane ruffles suggested that SNX18 is likely involved in the actin-driven remodeling of the plasma membrane which promotes formation of the nascent SCV.

### SNX18 functions as a scaffold for molecular machinery driving formation and scission of the nascent SCV

**Figure 3:**
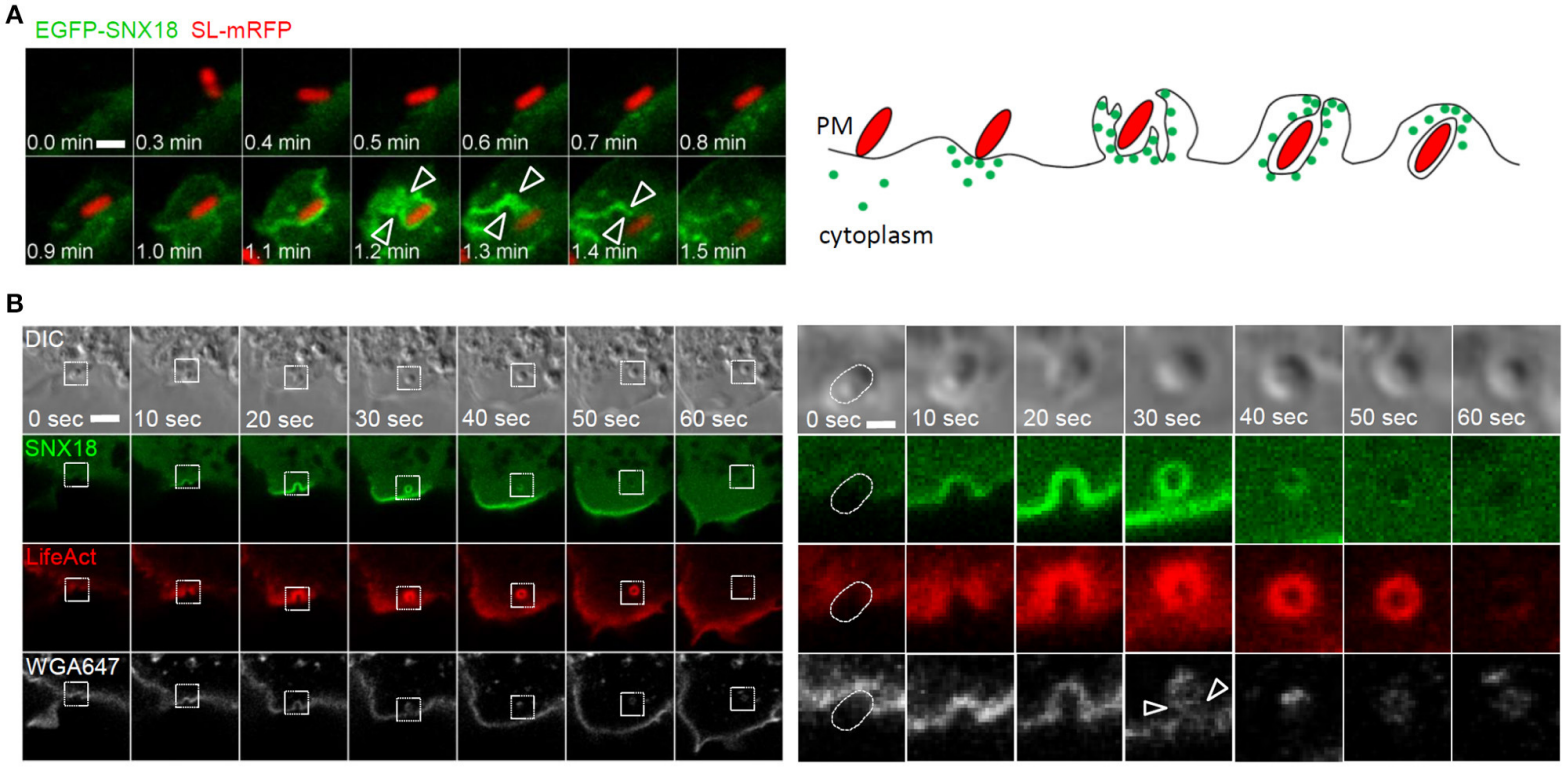
The recruitment of SNX18 to the site of SCV formation correlates with a burst of actin polymerization and membrane scission: **(A)** Series of confocal sections from a time-lapse of EGFP-SNX18-expressing cells infected with SL-mRFP. SNX18 relocates from cytosol to *Salmonella*-induced membrane ruffles at the site of bacterial attachment with a transient accumulation of SNX18 during the SCV scission from the plasma membrane (arrowheads). Bar = 2 μm. The diagram illustrates SNX18 (green dots) localization during bacterial internalization and at the early stage of the SCV formation. **(B)** Series of confocal sections from a time-lapse of HEK293 cells coexpressing EGFP-SNX18 with LifeAct-Ruby and infected with SL-mRFP in the presence of wheat germ agglutinin coupled with Alexa Fluor 647 (WGA-647). The burst of SNX18 and LifeAct-Ruby probe during bacteria internalization and SCV scission (indicated by arrowheads) and their depletion after the SCV detachment from the plasma membrane. Entering bacteria are visible in DIC. The panel on right shows enlarged details from the left panel. Bars = 10 μm (left panel) or 2 μm (right panel).

The dynamics of SNX18 recruitment to the site of bacteria entry was next determined by live imaging at a higher spatio-temporal resolution. We observed a rapid increase in SNX18 at the site of bacterial docking, followed by recruitment of SNX18 to the edge of extending membrane ruffles that folded back to enclose bacteria, and continuous accumulation of SNX18 was then detected in the membrane of the nascent SCV during its formation and scission from the plasma membrane. The local increase in EGFP-SNX18 fluorescence density was detected within 10–20 s following bacterial contact with the cell surface and the process of internalization and SCV scission was completed within 1 min. Subsequently, SNX18 rapidly depleted from the fully internalized SCV (Figure 3A and Movie 2).

Next we addressed the possibility that SNX18 functions in actin-driven membrane reorganization leading to bacterial confinement within closing membrane ruffles and scission of the nascent SCV from the plasma membrane. To visualize actin dynamics during bacteria internalization, the EGFP-SNX18 expressing cells were transfected with LifeAct-Ruby, a fluorescent 17-amino acid peptide which detects filamentous actin (F-actin) structures in live cells (Riedl et al., 2008), and infected with non-fluorescent *S*. Typhimurium in the presence of fluorescently coupled wheat germ agglutinin, which selectively binds to plasma membrane. Live imaging of these cells revealed that upon contact of bacteria with the cell surface, the burst of SNX18 correlates with a burst of actin polymerization during closure of the membrane ruffle around the nascent SCV and during SCV scission from the plasma membrane (within the first min of bacteria internalization). Once the SCV was fully internalized, SNX18 rapidly dissociated from the SCV followed by depolymerization of the F-actin coat (Figure 3B).

**Figure 4:**
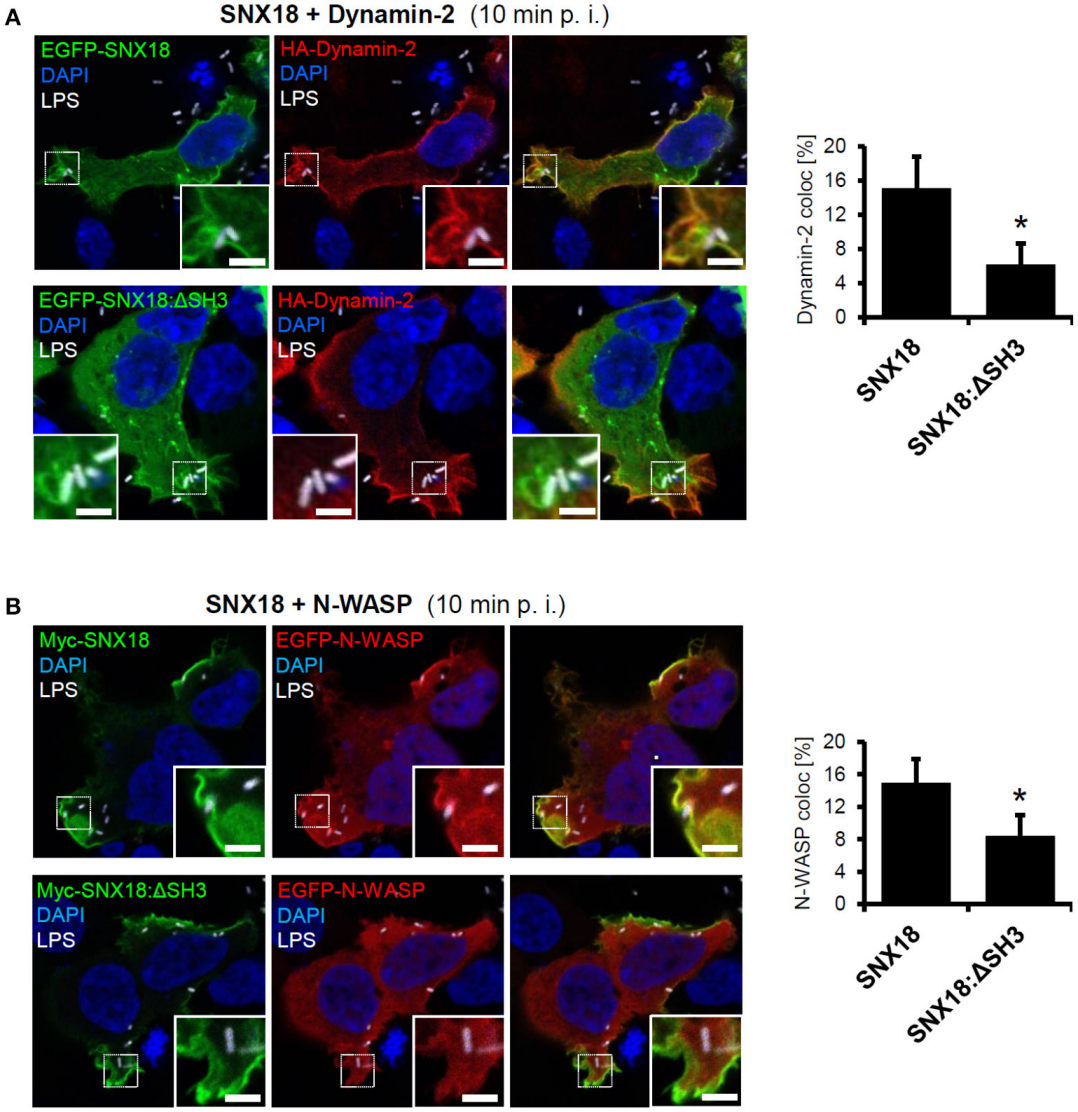
SNX18 functions as a scaffold for N-WASP and Dynamin-2 recruitment to the site of SCV formation. Co-localization between SNX18 and Dynamin-2 **(A)** or SNX18 and N-WASP **(B)** within *Salmonella*-containing membrane ruffles is significantly reduced in cells expressing EGFP-SNX18 construct lacking the SH3 domain. Cells co-expressing EGFP- or Myc-tagged SNX18 with HA-tagged Dynamin2 **(A)** or EGFP-N-WASP **(B)** fixed 10 min post infection (p. i.) and immunolabeled with anti-myc or anti-HA antibody followed by secondary antibody coupled to Alexa Fluor 546. Bacteria were detected by anti-LPS antibody followed by Alexa Fluor 405 secondary antibody and DNA was stained with DAPI. The fluorescent images are pseudocolored as indicated. Bars = 5 μm. Co-localization of indicated proteins in bacteria-containing membrane ruffles was quantified. The ROIs were limited to bacteria-containing membrane ruffles (enlarged inserts) cropped to identical square size (12 × 12 μm). Co-localization is presented as a percentage of voxels which have both channel 1 and channel 2 intensities above threshold, expressed as a percentage of the total number of pixels in the image (including zero-zero pixels); *t*-test, ^*^p < 0.05; Between 10-20 ROI per sample were analyzed and bars indicate the mean + standard errors within typical experiment.

Recently it has been reported that SNX18 directly interacts with proteins required for endosome scission, Dynamin-2 and N-WASP (Park et al., 2010). Importantly, both of these proteins are also involved in the internalization of *S*. Typhimurium (Unsworth et al., 2004; Veiga et al., 2007). To determine whether *S*. Typhimurium can recruit Dynamin-2 and N-WASP to the site of invasion via SNX18, we analyzed colocalization of SNX18 with Dynamin-2 and N-WASP in cells during bacteria internalization. Substantial co-localization of SNX18 with both Dynamin-2 and N-WASP was found within bacteria-containing membrane ruffles and on the nascent SCV (Figures 4A,B). However, a significantly lower degree of co-localization was found in cells that co-expressed Dynamin-2 or N-WASP with SNX18 mutant lacking the N-terminal SH3 domain (SNX18:ΔSH3). Although the SNX18:ΔSH3 was recruited into *Salmonella*-induced membrane ruffles within 10 min of infection to a similar extent as the full length SNX18, its co-localization with Dynamin-2 and N-WASP was markedly reduced as shown on quantification of the co-localization within bacteria-containing membrane ruffles (Figures 4A,B).

Together, these results provide the evidence that *S*. Typhimurium triggers rapid recruitment of cytosolic SNX18 to the site of invasion to function in the actin-driven formation and scission of the nascent SCV from the plasma membrane together with Dynamin-2 and N-WASP. We propose that the SH3 domain is dispensable for SNX18 targeting to the plasma membrane but is required for recruitment of Dynamin-2 and N-WASP to distinct membrane subdomains of membrane ruffles during formation and closure of the nascent SCV.

### Mutation of SNX18 impairs *S*. Typhimurium internalization

**Figure 5:**
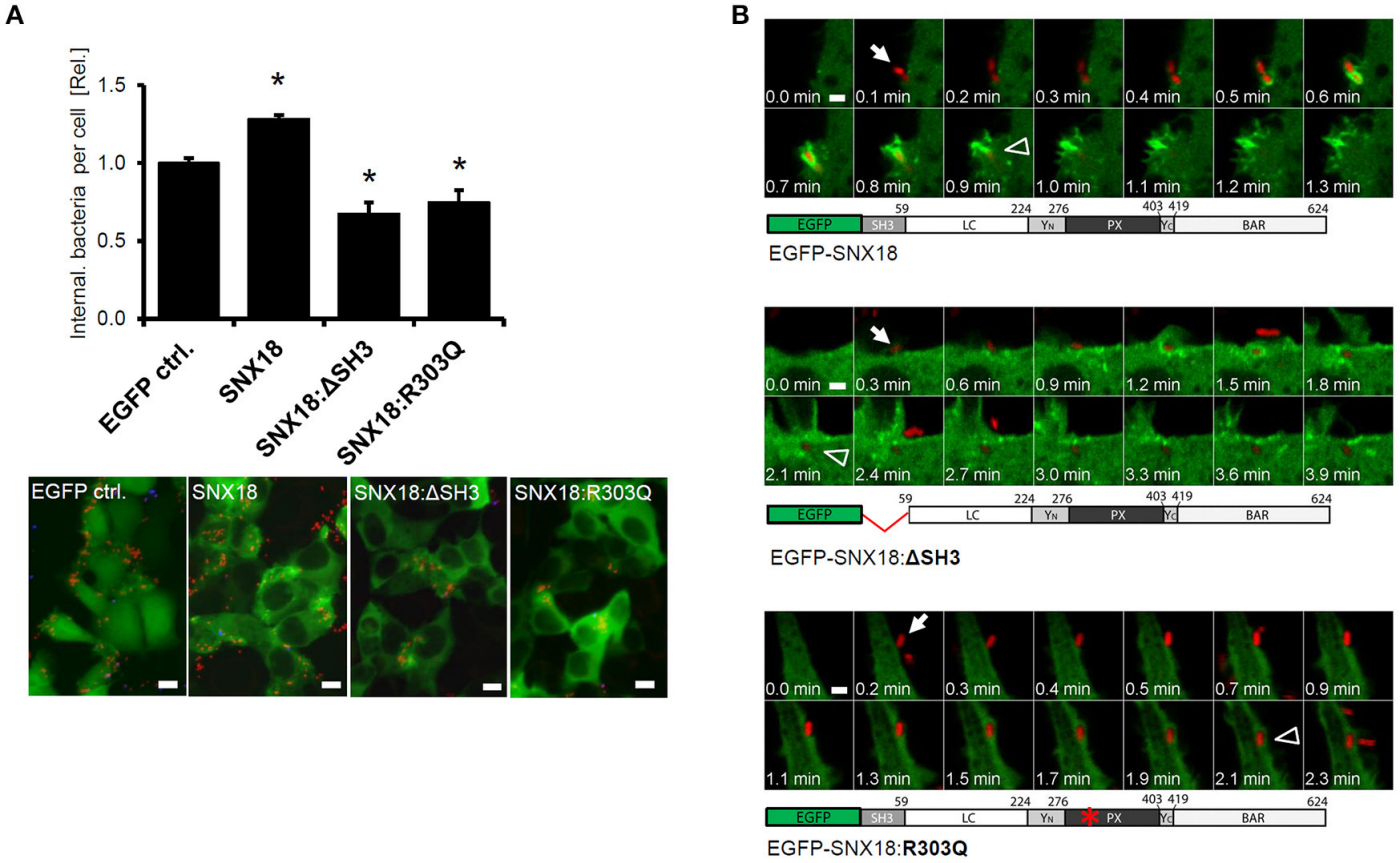
SNX18 requires the SH3 domain and an intact PtdIns-binding site to facilitate the uptake of the bacteria. **(A)** Quantification of bacteria internalization in cells overexpressing full length SNX18, SNX18 lacking the SH3 domain (SNX18:ΔSH3) or phosphoinositide binding mutant SNX18:R303Q in comparison to control cells (expressing EGFP only). Cells were fixed at 10 min post infection with SL-mRFP and intracellular bacteria were quantified by image analysis with exclusion of extracellular bacteria (immunolabeled with anti-LPS antibody). The average values in control cells were normalized to one to demonstrate the fold change in cells overexpressing SNX18 or the SNX18 mutants. ^*^p < 0.05. Bars indicate the mean + standard deviation between three experiments. Below: Examples of immunofluorescence images used for quantification. Bars = 10 μm. **(B)** Live imaging of HEK293 cells overexpressing EGFP-tagged SNX18, SNX18:ΔSH3, or SNX18:R303Q constructs and infected with SL-mRFP. Note the delay between bacteria first contact with the cell (arrows) and complete internalization (arrowheads) in cells expressing both SNX18 mutants and no sign of SNX18:R303Q recruitment to the site of bacteria invasion. Series of confocal sections from representative time-lapse are shown. Schematic diagrams of each construct are shown below. Bars = 2 μm.

The above results suggested that the SNX18 can mediate the process of *S*. Typhimurium internalization into host cells by acting as a scaffold for the recruitment of Dynamin-2 and N-WASP via its SH3 domain. Therefore, we next investigated whether transient overexpression of SNX18 mutants lacking the SH3 domain (SNX18ΔSH3) or phosphoinositide binding mutant (SNX18:R303Q) will affect the overall efficiency of bacteria internalization. The design of the phosphoinositide binding mutant was based on a structural study of SNX9 (a paralog of SNX18), which demonstrated that mutation of the corresponding arginine (Arg286) within α1 loop of phosphoinositide-binding pocket abrogates SNX9 binding to liposomes (Pylypenko et al., 2007). HEK293 cells were transfected with plasmids encoding wild-type or mutant SNX18 and infected with mCherry-expressing *S*. Typhimurium. The amount of internalized bacteria was determined by using a quantitative immunofluorescence assay. At 10 min post infection, cells overexpressing wild-type SNX18 exhibited a significantly higher number of internalized bacteria when compared with control cells. In contrast, a decrease in bacterial uptake was found in cells transfected with SNX18:ΔSH3 or SNX18:R303Q constructs relative to the control cells (Figure 5A). These results demonstrated that the efficiency of bacteria internalization is affected by loss-of-function mutations in SNX18.

To determine whether overexpression of SNX18:ΔSH3 and SNX18:R303Q mutants reduces bacteria internalization by interfering with the SCV formation, we analyzed the kinetics of this process by live cell imaging. The SNX18:ΔSH3 exhibited little difference in sub-cellular localization, recruitment to the site of bacterial internalization and transient association with the nascent SCV when compared to the full length SNX18 (Figure 4). In contrast, the phosphoinositide-binding mutant SNX18:R303Q remained exclusively cytosolic and exhibited no change during bacterial internalization. Although the internalization of bacteria was not blocked in cells expressing the SNX18 mutants, we frequently observed a delay between docking of the bacteria on the host cell and completion of the internalization process (scission of the SCV from the plasma membrane) in comparison to cells expressing the full-length SNX18 construct (Figure 5B and Movies 3–5).

Taken together, these data suggest that transient overexpression of SNX18:ΔSH3 or SNX18:R303Q may reduce the overall efficiency of *S*. Typhimurium internalization at the onset of infection, likely through defects or a delay in the recruitment of proteins involved in formation and/or scission of the nascent SCV. Because the uptake of bacteria in cells overexpressing the SNX18 mutants was only partially reduced, as we observed for the SNX18 knockdown cells, we propose that the SNX18-mediated pathway may represent one of the two independent but complementary invasion mechanisms recently described for *S*. Typhimurium (Hanisch et al., 2011) that are regulated either by SopB or by SopE/SopE2 effectors of SPI1-T3SS.

### Recruitment of SNX18 is triggered by *Salmonella* PtdIns-phosphatase SopB

Internalization of *S*. Typhimurium is mediated by orchestrated action of several SPI1-T3SS effectors, including SopB, SopD, SopE, SopE2, SptP, SipA, and SipC that disrupt tight junctions and cell polarity (Boyle et al., 2006), induce actin cytoskeleton remodeling (Cain et al., 2008) and promote SCV formation (Terebiznik et al., 2002; Hernandez et al., 2004; Bakowski et al., 2007). We therefore reasoned that the recruitment of SNX18 to the sites of bacterial invasion may be triggered by one or more of these effectors.

**Figure 6:**
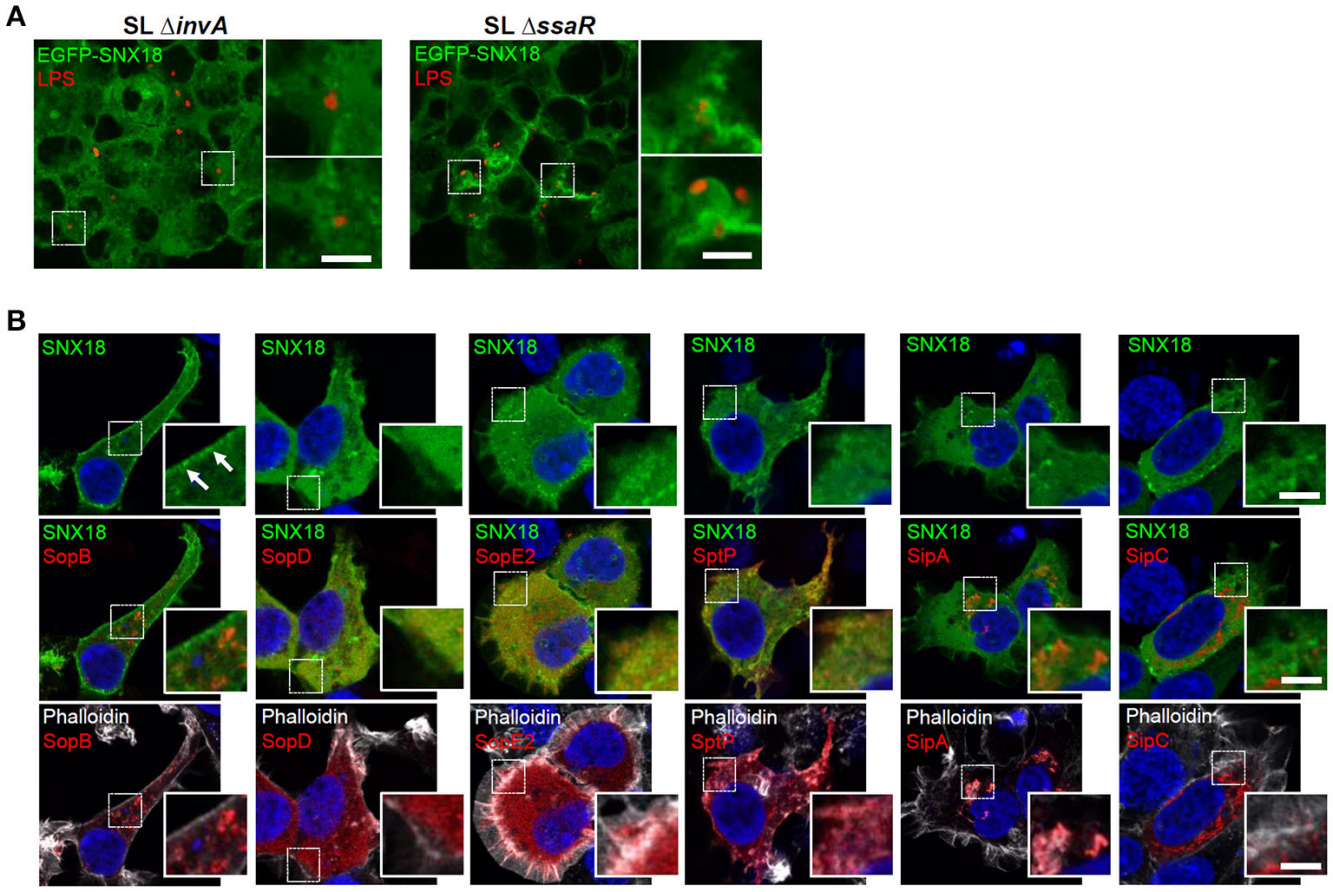
Activity of SPI1-T3SS effector SopB is necessary and sufficient for SNX18 recruitment to the plasma membrane. **(A)** SNX18 recruitment to the site of bacterial internalization is markedly perturbed in cells infected with the SPI1-T3SS inactive mutant Δ*invA* (left) while in cells infected with the SPI2-T3SS inactive mutant Δ*ssaR* (right) the burst of SNX18 at the site of bacterial internalization is comparable to that of induced by the wild type bacteria. Bacteria were detected by indirect immunofluorescence using anti-LPS antibody followed by secondary antibody coupled to Alexa Fluor 546. Bars = 5 μm. **(B)** Cells with stable expression of EGFP-SNX18 and transient (6 h) ectopic expression of myc-tagged SPI1-T3SS effectors with reported function in *S*. Typhimurium internalization. After fixation, the effectors were detected by anti-myc antibody followed by secondary antibody coupled to Alexa Fluor 546 (red), DNA was labeled with DAPI (blue), and F-actin was labeled by Phalloidin Alexa Fluor 647 (pseudocolored in white); Only expression of SopB resulted in depletion of cytosolic SNX18 and accumulation of SNX18 in the plasma membrane. Bars = 5 μm.

First we analyzed whether SNX18 mobilization in infected cells is dependent on activity of SPI1-T3SS and independent of SPI2-T3SS. The SPI1-T3SS deficient mutant of *S*. Typhimurium, Δ*invA*, is non-invasive and its contact with the EGFP-SNX18 expressing cells also failed to induce SNX18 relocation to the site of bacterial attachment at the cell surface. In turn, when cells were infected with the SPI2-T3SS deficient mutant, Δ*ssaR*, a burst of SNX18 was detected at the site of bacteria internalization to the extent similar to cells infected with the wild type bacteria (Figure 6A). To examine whether a particular SPI1-T3SS effector is sufficient to trigger SNX18 recruitment, we used transient ectopic expression of myc-tagged fusion proteins of individual SPI1-T3SS effectors in cells with stable expression of EGFP-SNX18. To minimize potential toxicity of effectors on the cell structure and functions, the cells were fixed and analyzed early at 4–6 h post transfection when the proteins were become detectable by indirect immunofluorescence. Our screen revealed that SNX18 retained its cytosolic localization except when co-expressed with SopB, where a substantial fraction of SNX18 accumulated in plasma membrane (Figure 6B). Although SNX18 remained cytosolic when co-expressed with the other SPI1 effectors, we did observe extensive membrane ruffles in SopE2-expressing cells and impaired actin cytoskeleton organization in cells expressing SipA, SipC, and SptP, which is consistent with the function of these effectors previously reported in the literature (Pizarro-Cerda and Cossart, 2004; Haglund and Welch, 2011).

**Figure 7:**
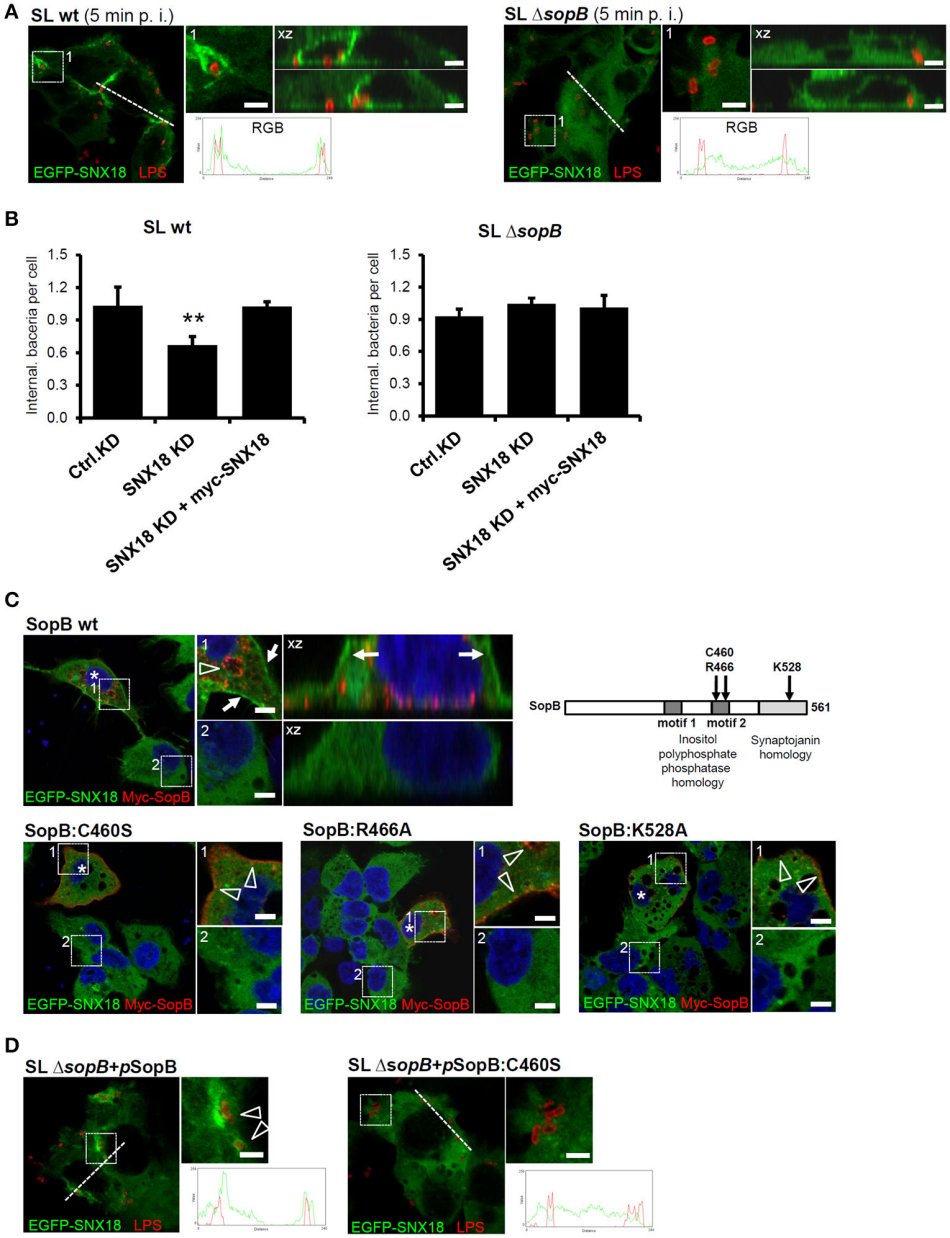
The SNX18-mediated invasion pathway of *S*. Typhimurium relies on inositol-phosphatase activity of SopB **(A)** *S*. *Typhimurium* mutant Δ*sopB* failed to trigger SNX18 recruitment to the site of invasion. EGFP-SNX18 expressing cells infected with wild type (left) or Δ*sopB* bacteria (right). Cells were fixed 10 min post infection (p. i.) and bacteria were labeled with anti-LPS antibody followed by secondary antibody coupled to Alexa Fluor 546 (red). The detail of *Salmonella*-containing ruffle (1) and the projection of confocal z-stack (xz) is shown together with the fluorescence intensity line profile (RGB) across the cell. Bars = 5 μm. **(B)** Quantification of the bacterial internalization (wild type and Δ*sopB*) in control knockdown cells, SNX18 knockdown cells and SNX18 knockdown cells transfected with myc-SNX18. Extracellular bacteria were detected by anti-LPS antibody followed by secondary antibody coupled with Alexa Fluor 405 and myc-SNX18 was detected by anti-myc antibody followed by secondary antibody coupled with Alexa Fluor 647. Numbers of internalized bacteria (mRFP-positive and LPS-negative) were counted by image analysis and average numbers per cell are shown. Bars indicate the mean + standard deviation between three experiments. **(C)** Localization of EGFP-SNX18 after ectopic expression of SopB or PtdIns-phosphatase defective mutants of SopB in non-infected cells. The wild type SopB localizes to endosomes and enlarged vacuoles (arrowheads) and induces SNX18 accumulation at the plasma membrane (arrows) whereas SopB with point mutation within the inositol polyphosphate 4-phosphatase homology (IPPH) motif 2 (C460S or R466A) or within the synaptojanin homology domain (K528A) localizes to the plasma membrane (arrowheads) and peripheral vesicles without visible changes to SNX18 localization. Cells were fixed 6 h post transfection, labeled with DAPI and myc-tagged SopB was detected by anti-myc antibody followed by secondary antibody coupled to Alexa Fluor 546 (red). Bars = 5 μm. **(D)** The failure of Δ*sopB* mutant bacteria to trigger SNX18 recruitment during internalization can be rescued by complementation using the full length SopB but not the inositol phosphatase deficient SopB:C460S. The HEK293 cells expressing EGFP-SNX18 were infected with the Δ*sopB* mutant bacteria transformed with pWSK29 plasmid encoding *sopB* or *sopB*:C460S. Immunofluorescence detection of bacteria and the RGB profiles were performed as in **(A)**, Bars = 5 μm. ^**^p ≤ 0.01.

To confirm that SopB is essential and sufficient for SNX18 recruitment to the sites of bacterial invasion, we next infected cells with an isogenic *sopB* mutant of *S*. Typhimurium (Δ*sopB* mutant). In comparison to the wild type *S*. Typhimurium, the Δ*sopB* mutant failed to recruit cytosolic SNX18 to the plasma membrane and to the site of bacterial internalization (Figure 7A). Moreover, quantification of bacterial internalization revealed that the reduction in numbers of internalized wild type *S*. Typhimurium (SLwt) in SNX18 knockdown cells can be restored by transient overexpression of myc-tagged SNX18. In contrast, numbers of internalized mutant (SLΔ*sopB*) were not significantly changed in SNX18 knockdown cells relative to control knockdown cells (Figure 7B). Together, these results further confirm that SNX18 functions in SopB-mediated internalization pathway of bacteria.

We next aimed to determine whether SNX18 recruitment was dependent on the ability of SopB to modify phosphoinositides. We constructed myc-tagged mutants of SopB that have been shown to abolish the phosphoinositide phosphatase activity of this effector: C460S, R466A, and K528A (Drecktrah et al., 2004). In cells co-expressing EGFP-SNX18 with wild type SopB, a substantial amount of SNX18 localized to the plasma membrane, while SopB localized primarily to enlarged endosomal structures most likely induced as a result of PtdIns(3)P accumulation at their membrane (Figure 7C). In contrast, all three SopB mutant proteins localized to the plasma membrane and small peripheral endosomal structures, suggesting they still can associate with the plasma membrane but fail to induce accumulation of enlarged PtdIns(3)P-positive endosomes. The co-expression of SopB mutant proteins did not alter the predominantly cytosolic localization of SNX18 and neither wild type myc-SopB nor the myc-tagged sopB mutant proteins co-localized with SNX18. Importantly, the failure of Δ*sopB* mutant bacteria to trigger SNX18 relocation to the membrane ruffles was fully restored when *sopB* was expressed from a plasmid in strain SLΔ*sopB*+pSopB. However, complementation was not achieved with introduction of plasmids encoding the inositolphosphatase-deficient mutant of SopB (C460S) (Figure 7D). Furthermore, SopB activates Akt/protein kinase B in epithelial cells infected by *S*. Typhimurium (Steele-Mortimer et al., 2000) and lack of Akt activation in cells infected with Δ*sopB* mutant was efficiently restored in cells infected with SLΔ*sopB*+pSopB strain (data not shown) what implies that the plasmid-encoded SopB in SLΔ*sopB* was efficiently translocated to infected cells.

Since SopB can also indirectly enhance Rho GTPase activation (Patel and Galan, 2006), we next examined whether SNX18 recruitment to the plasma membrane occurs independently or as a consequence of Cdc42 and/or Rac1 activation. Upon expression of constitutively active (CA) forms of Rac1 or Cdc42 in non-infected cells, Rac1, and Cdc42 localized to the plasma membrane and Rac1 induced formation of extensive membrane ruffles. However, in both cases SNX18 retained its cytosolic localization. Expression of dominant negative (GTPase-defective) forms of Cdc42 and Rac1 did not inhibit SNX18 recruitment to the site of bacterial invasion (Figure S2) and inhibition of Rac1-GTPase activity by specific, cell-permeable and reversible inhibitor NSC23766 did not perturb SNX18 recruitment to the site of bacteria internalization (Figure S3).

Collectively, these results demonstrate that relocation of SNX18 to *Salmonella*-induced membrane ruffles occurs independently of Cdc42 and Rac1 activation and that the PtdIns-phosphatase activity of SopB is necessary to drive SNX18 recruitment to the site of bacteria invasion. However, SNX18 and SopB probably do not directly interact, in agreement with our co-immunoprecipitation experiments detecting no interaction between SNX18 and ectopically expressed SopB (not shown).

### SNX18 recruits to membrane subdomains enriched in PtdIns(3,4)P2

Given that the phosphoinositide binding of SNX18 is essential for membrane recruitment, and this recruitment is dependent on the PtdIns-phosphatase activity of SopB, we next investigated whether SNX18 is recruited to the plasma membrane subdomains in response to SopB-mediated manipulation of phosphoinositide composition, since it has been previously reported that SopB is essential for local increase in PtdIns(3,4)P2 and PtdIns(3,4,5)P3 and depletion of PtdIns(4,5)P2 at *Salmonella*-induced membrane ruffles (Terebiznik et al., 2002; Mason et al., 2007; Mallo et al., 2008).

To visualize the local enrichment or depletion of particular membrane-bound phosphoinositides at the site of bacterial invasion, EGFP-tagged fusion proteins with (i) FERM domain of Ezrin, (ii) Pleckstrin Homology (PH) domain of Akt (also known as Protein Kinase B), or (iii) 2xFYVE domain of mouse Hours protein (hepatic growth factor-regulated tyrosine kinase substrate) were expressed in HEK293 cells. The FERM-EGFP recognizes and binds to PtdIns(4,5)P2 while EGFP-AKT-PH has affinity to PtdIns(3,4)P2 and/or PtdIns(3,4,5)P3 and 2xFYVE-EGFP has been used as a highly selective probe of PtdIns(3)P (James et al., 1996; Barret et al., 2000; Pattni et al., 2001). Following infection of these cells with mRFP-expressing bacteria, the FERM-EGFP with exclusive plasma membrane localization became markedly enriched at the cell periphery likely in response to an increase in membrane ruffling triggered by invading bacteria. However, closer inspection of membrane ruffles revealed a substantial local depletion of FERM-EGFP at the sites of bacterial internalization within these membrane subdomains and no association of FERM-EGFP was detected on fully internalized SCV (Figure S4). The EGFP-AKT-PH construct localized preferentially to the plasma membrane and to *Salmonella*-induced ruffles and exhibited substantial increase in fluorescence density around bacteria during the SCV formation but not around fully internalized SCV (Figure S4). In cells expressing the 2xFYVE-EGFP, the construct localized to tubular endosomal structures, exhibited a limited recruitment to the site of bacterial invasion, but associated markedly with fully internalized SCV (Figure S4). These results demonstrate that a transition occurs between accumulation of PtdIns(3,4)P2/PtdIns(3,4,5)P3 and PtdIns(3)P on the membrane of the SCV during early steps of the organelle biogenesis, which is in agreement with previous reports (Pattni et al., 2001; Mallo et al., 2008).

**Figure 8:**
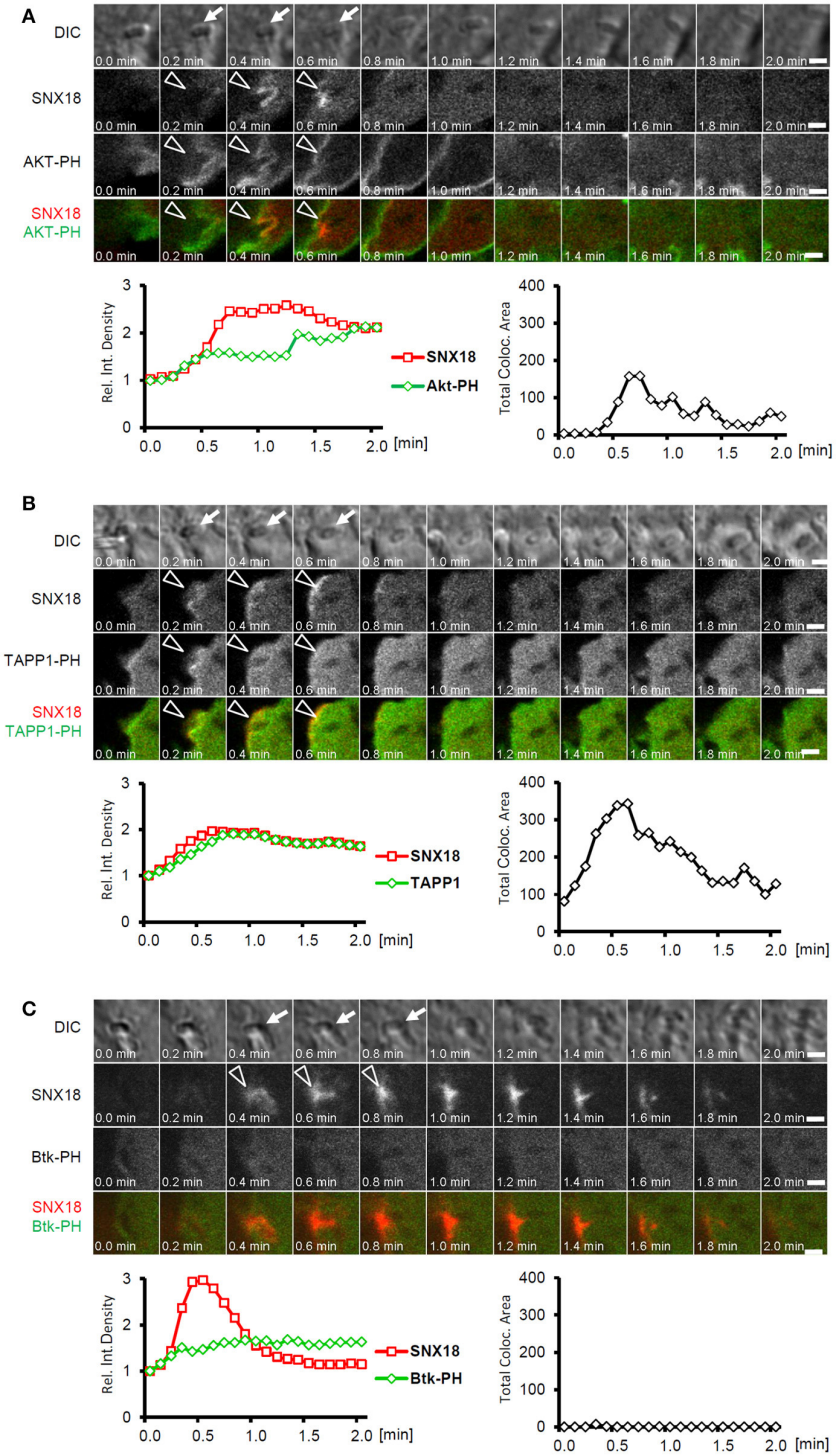
SNX18 is recruited to PtdIns(3,4)P2-enriched membrane subdomains. Correlation between recruitment kinetics of SNX18 and that of Akt-PH **(A)**, TAPP1-PH **(B)**, and Btk-PH **(C)** during bacteria internalization; Panels show series of still images from time-lapse movie of cells co-transfected with mCherry-SNX18 and Akt-PH-EGFP **(A)**, TAPP1-EGFP **(B)**, and Btk-PH-EGFP **(C)** and infected with non-fluorescent bacteria (indicated by arrows in DIC channel); Simultaneous recruitment of SNX18 and Akt-PH or TAPP1 to the site of bacterial internalization is indicated by arrowheads; Below: Correlation of transient increase in fluorescent density between mCherry and EGFP (left) were analyzed from sequence of 21 images (values at T = 0 were normalized to 1 to show a relative increase/decrease over time); Time profile of total co-localization area between mCherry and EGFP signal (right); Bars = 5 μm. Shown are results from a typical live imaging experiment.

To determine whether SNX18 recruits simultaneously to the same membrane subdomains as EGFP-AKT-PH construct, we monitored the dynamics of EGFP-AKT-PH and mCherry-SNX18 recruitment in live cells during the *S*. Typhimurium internalization. A substantial amount of EGFP-AKT-PH localized to the plasma membrane prior to infection, yet a distinct increase in EGFP fluorescence intensity was detected at the site of bacterial internalization. Moreover, kinetics of relative increase in fluorescence intensity indicated that Akt-PH and SNX18 recruited simultaneously to bacteria-induced membrane ruffles within the first 30 s of bacteria internalization (Figure 8A and Movie 6). Since the EGFP-AKT-PH probe does not distinguish between PtdIns(3,4)P2 and PtdIns(3,4,5)P3, we next aimed to determine whether SNX18 exhibits selective affinity to one of these phosphoinositides. For this, we co-expressed mCherry-SNX18 with EGFP-tagged PH domain of Tandem PH-domain containing protein (TAPP1) or with EGFP-tagged PH domain of Bruton's tyrosine kinase (Btk). The PH domain of TAPP1 possesses specific affinity to PtdIns(3,4)P2 (Dowler et al., 2000), while the PH domain of Btk binds exclusively to PtdIns(3,4,5)P3 (Salim et al., 1996). The SNX18 and TAPP1-PH became simultaneously enriched at the site of bacterial docking and internalization and a substantial increase in co-localization was detected during this process (Figure 8B and Movie 6). In contrast, we detected little increase in Btk-PH at the site of bacterial invasion and co-localization with SNX18 was below detection level (Figure 8C and Movie 6). Moreover, in cells overexpressing SNX18 together with AKT-PH or TAPP1-PH constructs the burst of SNX18 recruitment at the plasma membrane triggered by invading bacteria was notably attenuated in comparison to cells expressing mCherry-SNX18 alone or together with the Btk-PH-EGFP. These data suggest that SNX18, TAPP1-PH and AKT-PH can compete for binding to PtdIns(3,4)P2 and that SNX18 is recruited to membrane subdomains selectively enriched in PtdIns(3,4)P2.

Since we observed the simultaneous burst of SNX18 and Akt within *Salmonella*-induced membrane ruffles, we were interested whether SNX18 recruitment is dependent on the activation of Akt, which can also be triggered by the inositol phosphatase activity of SopB (Steele-Mortimer et al., 2000). Pre-treatment of cells with Akt-specific inhibitor AKTi1/2 did not markedly perturb recruitment of EGFP-SNX18 to the site of bacteria invasion, while the phosphorylation of Akt elicited by wild type *S*. Typhimurium (but less by Δ*sopB* mutant) at 10 min post infection was completely abolished (data not shown).

Taken together, these results demonstrate that SNX18 is recruited to *Salmonella*-induced membrane invaginations in response to SopB-mediated accumulation of PtdIns(3,4)P2 and that SNX18 recruitment occurs together with Akt but independently of Akt activation.

## Discussion

*S*. Typhimurium orchestrates its internalization into host cells through the action of multiple SPI1-T3SS effectors, which hijack the host macropinocytosis pathway for bacterial uptake. A critical step in this pathway is the formation and scission of the nascent SCV mediated by dynamin-2 and N-WASP, however the precise mechanism of the recruitment of these proteins to the sites of bacterial invasion has not been described. Here we identify a function for SNX18 in this process and demonstrate that engagement of SNX18 occurs via the action of the *Salmonella* SPI1-T3SS effector SopB. The recruitment of SNX18 to the plasma membrane is induced by the expression of SopB alone and SopB's phosphatidylinositol phosphatase activity is essential for the spatial and temporal recruitment of SNX18 induced during *S*. Typhimurium invasion. Furthermore, mutation of the phosphoinositide binding site within the PX domain of SNX18 completely abolished SNX18 recruitment to the plasma membrane. We propose that activated SNX18 binds to phosphoinositides locally modified during *Salmonella* invasion and functions as a membrane scaffold for the recruitment of the molecular components of actin-driven membrane remodeling. This study identified SNX18 as a novel target of *S*. Typhimurium and we propose that in contrast to other sorting nexins that have been previously linked to the SCV maturation, SNX18 is engaged at the onset of bacterial internalization to function in biogenesis of membrane ruffles and macropinocytosis. Hereby *S*. Typhimurium recruits SNX18 and its associated proteins via inositol-phosphatase activity of SopB to locally and transiently upregulate macropinocytosis and thus facilitate invasion of the host cell. Nevertheless, the invasion process is—in parallel—orchestrated and dependent on additional *Salmonella* effector proteins including SopE and SopE2 which allows *S*. Typhimurium to fine-tune the efficiency of invasion into various cell types and/or via multiple endocytic pathways. Like SNX18, SNX9 is recruited to the site of *Salmonella* invasion, via its PX domain, and that the capacity of *Salmonella* to invade cells with a transient siRNA knock-down of SNX9 is reduced (Piscatelli et al., 2016). Therefore, it appears that SNX18 and SNX9 may have redundant functions during *Salmonella* invasion.

Studies into *Salmonella* internalization have been complicated by an array of mechanisms these bacteria have evolved to invade macrophages and polarized or non-polarized epithelial cells, and to activate or suppress various host cell signaling pathways. Thus, blocking of a single pathway often results in insignificant changes in the ability of *Salmonella* to invade cells. So far, only cytochalasin D has been found to completely block *S*. Typhimurium internalization in both epithelial cells and macrophages (Finlay et al., 1991; Forsberg et al., 2003), highlighting the indispensable role of actin cytoskeleton remodeling for bacteria entry. Moreover, two independent but complementary mechanisms of SopE-dependent and SopB-mediated internalization pathways of *S*. Typhimurium have been described (Stender et al., 2000; Hanisch et al., 2011). The first is characterized by the activation of Rac1/Cdc42 followed by activation of WAVE and WASH complex and Arp2/3-mediated actin polymerization, leading to extensive membrane ruffle formation, while the second is mediated by Rho kinase and Myosin II activity and occurs through plasma membrane invaginations without membrane ruffling (Stender et al., 2000; Hanisch et al., 2011). Indeed, we observed that bacteria entered the cell by two morphologically distinct mechanisms, but how these mechanisms differ at the molecular level is difficult to discern because both pathways seems to be utilized when multiple bacteria infect a single cell.

We found that cells overexpressing SNX18 exhibited a considerably increased rate of macropinocytosis, whereas the impact on *S*. Typhimurium internalization was relatively mild. Similarly, in SNX18 knockdown cells, the rate of macropinocytosis was reduced to a greater extent than internalization of bacteria. We argue that since *S*. Typhimurium infection elevates macropinocytosis via the action of translocated effectors, any additional gain from the direct expression of SNX18 might be limited. The fact that we observed only partial reduction in bacterial internalization efficiency in SNX18 knockdown cells may reflect the ability of bacteria to trigger the internalization process via a complementary mechanism which can be independent of SopB and SNX18. It will be interesting to find whether *S*. Typhimurium utilizes multiple pathways simultaneously or possesses an ability to fine-tune the invasion efficiency according to environmental cues. Nevertheless, the reduced invasiveness of SopB-deficient mutant of *S*. Typhimurium strain SL1344 or that of the wild type of SopE-deficient strain ST12023, both reported in the literature (Bakowski et al., 2007; Clark et al., 2011), suggests that the ability of these bacteria to trigger and utilize multiple internalization mechanisms enhances the net invasion efficiency. Thus, although we do not question that *S*. Typhimurium can utilize complementary pathway(s), our study describes the role of SNX18 in SopB-dependent invasion mechanism which significantly facilitates bacterial internalization.

The cells overexpressing the SNX18 mutant ΔSH3 or R303Q exhibited a mild but significant decrease in bacterial invasion yet the mechanism by which they interfere is unclear. The most likely explanation is that upon recruitment to the plasma membrane, the SNX18:ΔSH3 can interact with the endogenous pool of SNX18 resulting in production of a higher amount of SNX18 dimers with a reduced functionality. In contrast, similar pseudo-dimer formation between SNX18:R303Q and endogenous SNX18 would induce partial cytosolic retention of SNX18. Indeed, we did not detect any increase in fluorescence intensity of SNX18:R303Q at the site of bacterial internalization or at any other membrane subdomains. Nevertheless, Sorting nexin 9, 18, and 33 can readily be found as homodimers when isolated from cell lysates. Formation of heterodimers between SNX9-SNX18 and SNX9-SNX33 *in vitro* has been previously reported (Zhang et al., 2009; Park et al., 2010), but not confirmed by a recent study (van Weering et al., 2012) and ourselves (data not shown), and whether possible heterodimers represent functional units in intact cells *in vivo* still remains to be determined.

Interaction between SNX18 and membrane-bound phosphoinositides is essential for regulation of SNX18 function, yet the phosphoinositide specificity of SNX18 PX domain remains unclear. Filter binding assays and liposome co-sedimentation experiments using a GST fusion of SNX18 PX domain or PX-BAR module identified affinity for PtdIns(3,4)P2, PtdIns(3,5)P2, and PtdIns(4,5)P2 (Haberg et al., 2008; Nakazawa et al., 2011) and similar broad lipid specificity has also been reported for SNX9 (Shin et al., 2008; Yarar et al., 2008). However, the specificity of SNX18 and/or SNX9 for individual phosphoinositides in live cells is unknown. Using live cell imaging, our study provides insight into the recruitment of SNX18 to membrane subdomains enriched in PtdIns(3,4)P2 and highlights the importance of cell context in this assay because the spatio-temporal aspects of recruitment or depletion of phosphoinositide binding proteins is difficult to characterize by biochemical approaches. It is important to note that even transient overexpression of phosphoinositide sensing probes may imbalance the phosphoinositide metabolism by irreversible binding of these probes to particular phosphoinositide subspecies. This, as our results suggest, would also lead to affinity-dependent competition between SNX18 and the probes for binding PtdIns(3,4)P2. Nevertheless, our conclusions were based on comparison of recruitment kinetics between SNX18 and individual PtdIns-probes and the kinetics seemed to be comparable to that of cells expressing SNX18 without the PtdIns-probes.

Identification of SNX18 as a host cell factor involved in SopB-mediated formation of the nascent SCV at the plasma membrane builds upon the previously described role of SopB (formerly SigD) in hydrolysis of PtdIns(4,5)P2 during this process (Terebiznik et al., 2002). Terebiznik et al. (2002), demonstrated that the break-down of PtdIns(4,5)P2 in cells expressing SigD/SopB reduced the rigidity of the plasma membrane, suggesting that local PtdIns(4,5)P2 depletion may increase susceptibility of membrane for curvature and fission (Terebiznik et al., 2002). Therefore, besides the phosphoinositide sensing PX domain, the curvature-sensing BAR domain of SNX18 is likely equally important for SNX18 recruitment to membrane invaginations and its (indirect) function in the SCV scission.

It has been reported that SopB may act on several substrates *in vitro* including PtdIns(3,4)P2, PtdIns(3,5)P2, PtdIns(4,5)P2, and PtdIns(3,4,5)P3 (Norris et al., 1998; Marcus et al., 2001). However, a more recent study has shown that concentration of PtdIns(3,4)P2 and PtdIns(3,4,5)P3 in *S*. Typhimurium invasion ruffles actually increases, likely by activation of class II PI3-kinases via PtdIns(5)P as a product of SopB-mediated hydrolysis of PtdIns(4,5)P2 (Mallo et al., 2008). We propose that the transient local increase in PtdIns(3,4)P2 may be the driving cue for recruitment of SNX18, together with other phosphoinositide sensing factors like Akt. Plasma membrane recruitment of Akt is coupled with activation by phosphorylation (Steele-Mortimer et al., 2000; Marcus et al., 2001), but whether SNX18 requires activation for relocation or becomes activated upon plasma membrane recruitment still remains to be determined. A possibility remains open that to trigger SNX18 relocation the sole increase in PtdIns(3,4)P2 is not sufficient and may require an additional stimulus linked directly or indirectly to the activity of SopB.

It has been shown that upon delivery to the host cell the SopB targets SH3-containing GEF and thus can indirectly activate RhoG (Patel and Galan, 2006) that promotes Rac1 activation and membrane ruffling. Although we demonstrate that SopB-mediated recruitment of SNX18 to the plasma membrane occurs independently or upstream of Rac1 activation (Figures S2, S3) together with recruitment of Dynamin-2 and N-WASP, it remains to be determined whether SNX18 or SopB are also involved in activation of the GTPase activity of Dynamin-2 or in N-WASP activation of Arp2/3 machinery.

The study we present extends our understanding of *S*. Typhimurium-driven exploitation of the host molecular machinery that allows it to invade and replicate within epithelial cells. We propose that SNX18 and/or other sorting nexins are also involved in the uptake of other bacterial pathogens. It has also been reported that Enteropathogenic *Escherichia coli* (EPEC) secreted protein F (EspF) can directly interact with the SH3 domain of SNX9 and N-WASP to promote EPEC invasion via N-WASP/Arp2/3–mediated actin nucleation (Marches et al., 2006; Alto et al., 2007). However, EPEC induces a local increase of PtdIns(3,4,5)P3 at the site of attachment indirectly via recruitment of phosphatidylinositol 3-kinase in a translocated intimin receptor (Tir)-dependent manner (Sason et al., 2009). Therefore, it is possible that EPEC may recruit SNX9 by an indirect mechanism similar to that of SopB-mediated recruitment of SNX18. In relation to this, the close *Salmonella* relative *Shigella flexneri* utilizes a SopB homolog (IpgD) for activation of PI3K/Akt-survival (Pendaries et al., 2006). IpgD possesses a similar activity *in vitro* and its intact inositol 5'-phosphatase-homology motif is essential for Akt activation (Marcus et al., 2001). During *S. flexneri* invasion into epithelial cells IpgD elevates the level of ruffling and macropinoscytosis in cells. However, *S. flexneri* does not enter cells via these induced macropinosomes but rather uses a mechanism that creates a bacterium containing vacuole (BCV) in which the host membrane is always tightly associated with the bacteria during invasion (Weiner et al., 2016). The role of induced macropinosomes is required during the rupture of BCV to release *S. flexneri* into the cytoplasm (Weiner et al., 2016). Whether *Shigella* or other bacterial pathogens have developed a similar strategy to exploit the endocytic machinery of the host cell by recruitment of scaffolding proteins from SH3-PX-BAR sorting nexin family remains to be verified.

## Author contributions

All authors contributed to the conception, design, interpretation of the findings, drafting, and preparation of the manuscript. DL, XQ, YZ, and TB executed the experiments.

### Conflict of interest statement

The authors declare that the research was conducted in the absence of any commercial or financial relationships that could be construed as a potential conflict of interest.

## Supplementary material

Movie 1 SNX18 (EGFP) recruitment from cytosol to the plasma membrane during *S*. Typhimurium (mRFP) invasion. Zoom: Transient SNX18 recruitment to the nascent SCV in infected cell (1) and to the nascent macropinosomes in non-infected cell (2). HEK293 cells with stable expression of EGFP-SNX18, 1-5 min post infection. (0.1 min between frames, 7 fps).

Movie 2 SNX18 (EGFP) recruits to the edge of *Salmonella*-induced membrane ruffles and accumulates on the nascent SCV during its scission from the plasma membrane. HEK293 cells with stable expression of EGFP-SNX18, 1-5 min post infection. (0.1 min between frames, 7 fps).

Movie 3 SNX18 (EGFP) recruits to the site of *S*. Typhimurium (mRFP) attachment at the plasma membrane and accumulates during bacteria internalization into the nascent SCV. HEK293 cells, 16 h post transfection, 1–5 min post infection. (0.1 min between frames, 7 fps).

Movie 4 SNX18:ΔSH3 (EGFP) recruits to the site of *S*. Typhimurium (RFP) attachment at the plasma membrane and accumulates during bacteria internalization into the nascent SCV. HEK293 cells, 16 h post transfection, 1–5 min post infection. (0.3 min between frames, 7 fps).

Movie 5 SNX18:R303Q (EGFP) remains cytosolic during *S*. Typhimurium (RFP) attachment to the plasma membrane and prolonged internalization into the nascent SCV. HEK293 cells, 16 h post transfection, 1–5 min post infection. (0.1 min between frames, 7 fps).

Movie 6 Transient burst of SNX18 (mCherry) at the site of *S*. Typhimurium internalization occurs simultaneously with the Akt-PH and TAPP1-PH (EGFP), whereas no increase in Btk-PH (EGFP) signal was detected. HEK293 cells, 8 h post co-transfection, 1–5 min post infection. (0.2 min between frames, 7 fps).

## Abbreviations

SNX18: Sorting nexin 18
*S*. Typhimurium: *Salmonella enterica* serovar *Typhimurium;*
SL-mRFP: *S*. Typhimurium expressing mRFP
SCV: *Salmonella*-containing vacuole
*Salmonella* SPI1-T3SS: pathogenicity island 1—type three secretion system
PtdIns: Phosphatidylinositol
N-WASP: Neuronal Wiscott-Aldrich syndrome protein
MOI: Multiplicity of infection
PH domain: Pleckstrin homology domain.
