# Supplementary material for: SopB-Mediated Recruitment of SNX18 Facilitates Salmonella Typhimurium Internalization by the Host Cell
Source: Front Cell Infect Microbiol. 2017 Jun 15;7:257. doi: 10.3389/fcimb.2017.00257 (PMC5471308; doi:10.3389/fcimb.2017.00257)

Supplementary Information

Figure S1. SNX18 is enriched on plasma membrane-derived tubules during endosome scission from the plasma membrane. Clone selection was used to generate a cell line of HEK293 cells with stable expression of EGFP-SNX18. Confocal sections of live cell after 2 min incubation with 1μM lipophilic fluorescent dye FM4-64 (red), Left: A z-stack shows SNX18 in the cytosol and in association with plasma membrane-derived tubules enriched at the bottom of the cells (shown in detail 1); Right: The xz-projection and the fluorescent intensity line profile below indicates accumulation of EGFP-SNX18 around plasma membrane invaginations.

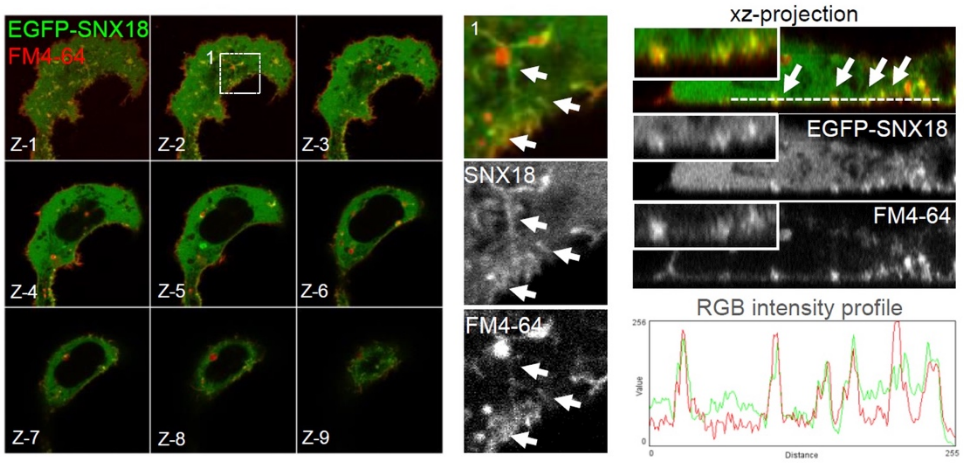

Figure S2. SopB-mediated recruitment of SNX18 to the plasma membrane is independent on activation of Rho-GTPases. Left panel: In non-infected cells expressing constitutively active (CA) forms of Rac1 or Cdc42, the Rac1 and Cdc42 localize to the plasma membrane, while SNX18 maintains the cytosolic localization. Note that expression of constitutively-active Rac1 induces formation of extensive membrane ruffles in non-infected cells without recruiting SNX18 (arrowheads). Right panel: Expression of dominant negative (DN) forms of Cdc42 or Rac1 (GTPase-defective) does not inhibit SNX18 recruitment during bacterial internalization. Cells were fixed 10 min p.i. and bacteria were labelled by anti-LPS antibody (Alexa fluor 405) on permeabilized cells. Note that expression of Cdc42:DN (but not of Rac1:DN) abolished bacteria internalization into the cell without perturbing *Salmonella*-induced SNX18 recruitment to the plasma membrane (arrows). Bars = 5 µm.

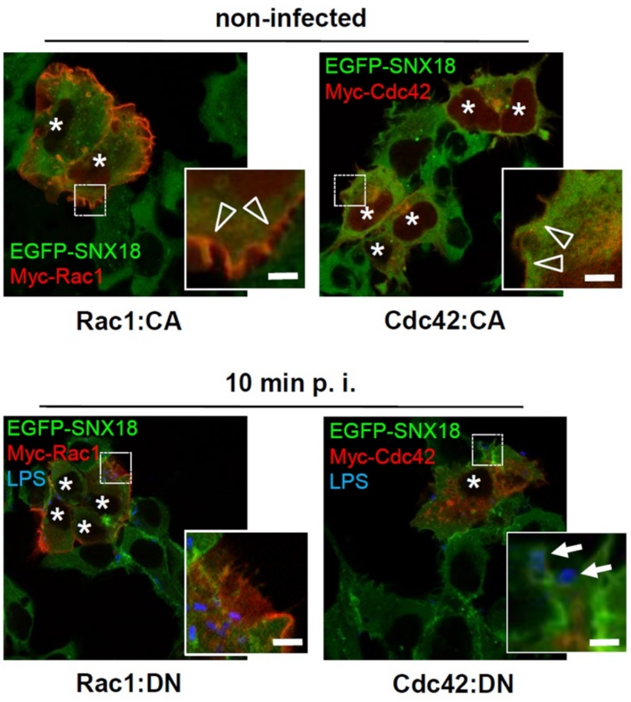

Figure S3. Inhibition of Rac1 GTPase does not perturb SNX18 recruitment to the site of bacteria invasion. HEK293 cells with stable expression of EGFP-SNX18 treated with 10 µM or 100 µM Rac1-specific inhibitor NSC23766 for 60 min pre-infection and then during 10 min of infection. Cells were fixed and labelled with phalloidin-TRITC and anti-LPS antibody (Alexa Fluor 405). Representative confocal sections are shown as merged RGB (top panels) and green channel (EGFP-SNX18) is shown separately in a grey scale (lower panels). Bars = 5 µm.

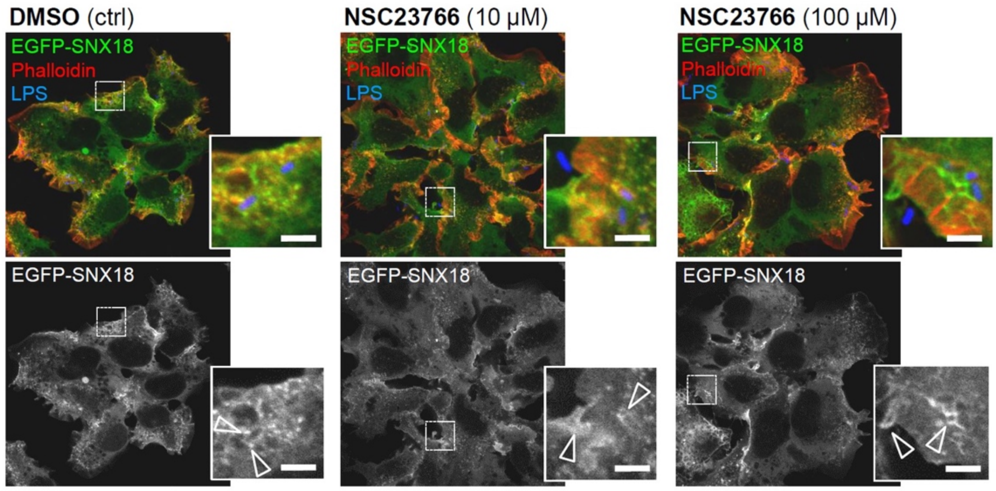

Figure S4. Recruitment of phosphoinositide binding probes to the site of bacteria invasion. The PtdIns(4,5)P2-binding FERM domain exhibits exclusive plasma membrane localization (1) but depletion from the site of bacteria internalization (2) and does not accumulate on the SCV (3). The PH domain of Akt with affinity to PtdIns(3,4)P2 and PtdIns(3,4,5)P3 localizes to the plasma membrane (1) but a distinct increase can be detected on the nascent SCV at the site of bacteria internalization (2) but not on fully internalized SCV (3). PtdIns(3)P-sensing 2xFYVE domain localizes to intracellular tubular vesicles (1), shows limited recruitment to the site of *S*. Typhimurium invasion (2) but discernable accumulation on fully internalized SCV (3). Cells were infected 12 hrs post transfection and fixed 10 min post infection with mRFP-expressing bacteria (wild type); confocal sections from representative images are shown (arrowheads indicate the SCV positive for SNX18); Bars = 5 μm

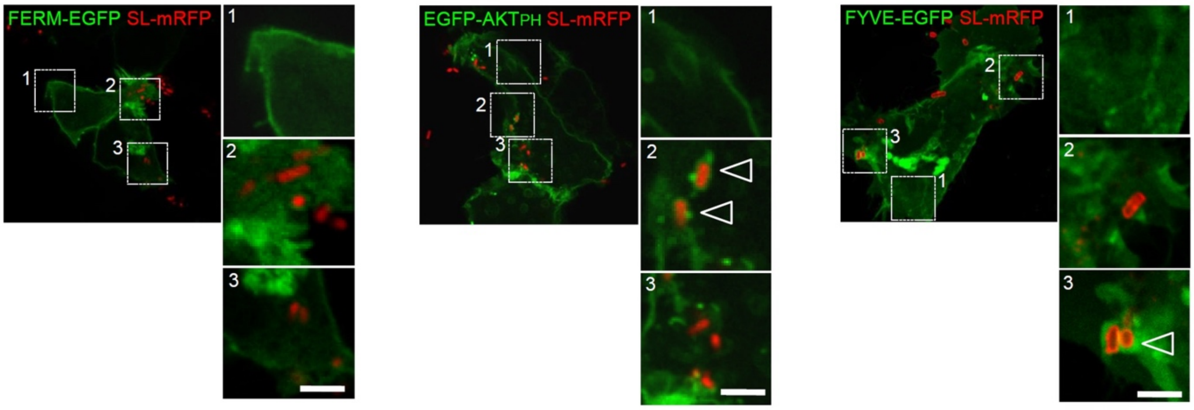

Supplement: Supplementary file 7 [file Presentation1.PDF]
